# Does ambient PM_2.5_ reduce the protective association of leisure-time physical activity with mortality? A systematic review, meta-analysis, and individual-level pooled analysis of cohort studies involving 1.5 million adults

**DOI:** 10.1186/s12916-025-04496-y

**Published:** 2025-11-28

**Authors:** Po-Wen Ku, Andrew Steptoe, Mark Hamer, Paola Zaninotto, Emmanuel Stamatakis, Ching-Heng Lin, Bin Yu, Ulla Arthur Hvidtfeldt, Xiang Qian Lao, Hsien-Ho Lin, Wei-Cheng Lo, Ole Raaschou-Nielsen, Shengzhi Sun, Linwei Tian, Su-Fen Wang, Yiqian Zeng, Yunquan Zhang, Shang-Ti Chen, Chien-Fong Huang, Yang Xia, Li-Jung Chen

**Affiliations:** 1 Graduate Institute of Sports and Health Management, National Chung Hsing University, Taichung, 402 Taiwan; 2 Department of Behavioural Science and Health, University College London, London, WC1E 6BT UK; 3 Institute of Sport Exercise and Health, Division of Surgery and Interventional Science, Faculty of Medical Sciences, University College London, London, WC1E 6BT UK; 4 Department of Epidemiology and Public Health, University College London, London, WC1E 6BT UK; 5 Charles Perkins Centre, Mackenzie Wearables Research Hub, Faculty of Medicine and Health, University of Sydney, Sydney, NSW 2006 Australia; 6 Department of Medical Research, Taichung Veterans General Hospital, Taichung, 407 Taiwan; 7 Institute of Applied Psychology, Tianjin University, Tianjin, China; 8 Academy of Medical Engineering and Translational Medicine, Tianjin University, Tianjin, China; 9 Danish Cancer Institute, Copenhagen, Denmark; 10 Department of Biomedical Sciences,, City University of Hong Kong, Hong Kong SAR, China; 11 Institute of Epidemiology and Preventive Medicine, National Taiwan University, Taipei, Taiwan; 12 Master Program in Applied Epidemiology, College of Public Health, Taipei Medical University, Taipei, Taiwan; 13 Department of Environmental Science, Aarhus University, Roskilde, Denmark; 14 School of Public Health, Capital Medical University, Beijing, 100069 China; 15 Beijing Municipal Key Laboratory of Clinical Epidemiology, Capital Medical University, Beijing, China; 16 School of Public Health, The University of Hong Kong, Hong Kong SAR, China; 17 Department of Geography, National Changhua University of Education, Changhua, 500 Taiwan; 18 School of Public Health, Wuhan University of Science and Technology, Wuhan, China; 19 Department of Tourism, Recreation, and Leisure Studies, National Dong Hwa University, Hualien, Taiwan; 20 Department of Clinical Epidemiology, Shengjing Hospital of China Medical University, Shenyang, China; 21 Department of Exercise Health Science, National Taiwan University of Sport, Taichung, 404 Taiwan; 22 Department of Psychosis Studies, Institute of Psychiatry, Psychology and Neuroscience, King’s College London, London, SE5 8AF UK

**Keywords:** Exercise, Air pollution, Air quality, Death, Joint association, Combined effect

## Abstract

**Background:**

This study assessed whether higher levels of fine particulate matter (PM_2.5_) reduce the protective effects of leisure-time physical activity (LTPA) on all-cause, cardiovascular, and cancer mortality, and explored the PM_2.5_ threshold beyond which attenuation occurs.

**Methods:**

We conducted two complementary investigations. First, a systematic review and meta-analysis (per PRISMA guidelines) identified eligible cohort studies from PubMed, Web of Science, Embase, and SPORTDiscus (from inception to 6 January 2025) that examined the independent or joint associations of LTPA and PM₂.₅ with mortality among adults (≥ 18 years). Second, an individual-level pooled analysis using harmonized data from three cohorts was performed using Cox regression modeling to assess the associations observed in the meta-analysis.

**Results:**

In Study One, a total of seven cohort studies (*n* = 1,515,094; deaths = 115,196) were included in the meta-analysis, revealing that the reduction in all-cause mortality risk diminished with higher PM_2.5_ exposure. Meeting the recommended LTPA level (7.5–15 MET-h/week) reduced all-cause mortality risk by approximately 30% at PM_2.5_ < 25 μg/m^3^ but only 12–15% at 25 + μg/m^3^. Study Two (three cohorts; *n* = 869,038; deaths = 45,080) confirmed this pattern. Individuals meeting the recommended LTPA level (7.5–15 MET-h/week) had a lower risk of all-cause mortality compared to those in the highest-risk group (reference: < 1 MET-h/week and PM_2.5_: 35–50 μg/m^3^). Hazard ratios (HRs) varied by PM_2.5_ exposure, with lower HRs indicating a greater protective effect: 35–50 μg/m^3^ (HR = 0.75, 95% CI: 0.61–0.93), 25–35 μg/m^3^ (HR = 0.67, 95% CI: 0.57–0.79), 15–25 μg/m^3^ (HR = 0.34, 95% CI: 0.29–0.39), 10–15 μg/m^3^ (HR = 0.34, 95% CI: 0.28–0.41), and < 10 μg/m^3^ (HR = 0.30, 95% CI: 0.25–0.37). Higher levels of LTPA were generally associated with lower all-cause and cause-specific mortality across most PM₂.₅ exposure categories, but the protective effects were attenuated at PM₂.₅ levels 25 + μg/m^3^ for all outcomes and became non-significant for cancer mortality at 35–50 μg/m^3^.

**Conclusions:**

LTPA is beneficial for all-cause, cardiovascular, and cancer mortality even at relatively high PM_2.5_ levels, with greater benefits observed under cleaner air conditions. However, its protective effects are attenuated at 25 + μg/m^3^ for all outcomes and become less evident at 35–50 μg/m^3^, particularly for cancer mortality.

**PROSPERO Registration Number:**

CRD42023395364.

**Supplementary Information:**

The online version contains supplementary material available at 10.1186/s12916-025-04496-y.

## Background

Leisure-time physical activity (LTPA) is associated with a lower risk of morbidity and mortality [1, 2]. Nevertheless, participation in outdoor LTPA also leads to increased exposure to air pollutants, including fine particulate matter (PM_2.5_) [3, 4]. Long-term exposure to PM_2.5_ has been linked to elevated morbidity and mortality [5]. It was identified as the leading contributor to the global disease burden among 88 risk factors analyzed in 2021. It accounted for 8.0% (95% CI: 6.7–9.4) of total Disability-Adjusted Life Years (DALYs) worldwide [6]. Recent estimates suggest that PM_2.5_ was responsible for 4.48 million cardiovascular-related deaths globally [7]. Consequently, there is concern that the beneficial effects of LTPA on all-cause or cause-specific mortality (e.g., cardiovascular or cancer mortality) could potentially be offset by exposure to PM_2.5_ [8]. However, the current body of evidence from prospective cohort studies does not provide supporting evidence [9].

Eight cohort studies involving adults have been conducted across different regions, including the US [10, 11], the UK [12, 13], Hong Kong (Special Administrative Region of China) [14], and Taiwan (R.O.C.) [15–17], to investigate the potential link between LTPA and all-cause and cardiovascular mortality concerning PM_2.5_ exposure. These investigations collectively revealed that engagement in LTPA energy expenditure is associated with a decreased risk of mortality regardless of the levels of PM_2.5_ present. Little evidence was found to suggest that PM_2.5_ exposure attenuates the relationships between LTPA and all-cause or cause-specific mortality.

Notably, all these investigations were conducted within a single country, exposing participants to a relatively narrow range of annual mean PM_2.5_ concentrations (μg/m^3^), e.g., the UK (median = 9.9, interquartile range [IQR] = 1.3) [13], the USA (median = 10.6, IQR = 3.2) [11], Taiwan (median = 23.9, IQR = 6.2) [15], and Hong Kong (Median = 35.3, IQR = 3.4) [14]. Detecting the influence of PM_2.5_ on the LTPA-mortality relationship may be challenging when studies are conducted in areas characterized by a limited PM_2.5_ concentration range. Moreover, findings derived from studies conducted in regions with lower PM_2.5_ levels might not be directly applicable to areas with higher PM_2.5_ concentrations, and vice versa. Furthermore, these studies primarily focused on the general adult population, with less attention given to diverse population subgroups, including different sexes and individuals who are particularly susceptible to the adverse impacts of PM_2.5_ exposure (e.g., individuals with cardiovascular diseases, and older adults etc.) [18, 19].

This study aimed to bridge gaps in the existing literature by investigating (a) whether the associations of LTPA with all-cause mortality in adults aged 18 or older were attenuated by ambient PM_2.5_ levels, through a systematic review and meta-analysis encompassing a wide range of PM_2.5_ exposure levels; (b) if attenuation effects exist, identifying the specific PM_2.5_ concentration threshold above which such effects occur; and (c) whether these associations remain consistent across all-cause, cardiovascular, and cancer mortality in different sexes, age groups, and populations susceptible to harmful effects of PM_2.5_ using individual-level pooled analysis of cohort studies.

## Methods

### Study one: systematic review and meta-analysis

The systematic review and meta-analysis were conducted and reported following the guidelines of the Preferred Reporting Items for Systematic Reviews and Meta-Analyses (PRISMA) [20], and the protocol was registered with the PROSPERO (No.: CRD42023395364).

#### Data sources and search strategy

The first strategy involved conducting a systematic search of four electronic databases (MEDLINE, Web of Science, Embase, and SPORTDiscus) that was conducted from their inception to 6 January 2025. To find potential cohort studies examining the relationships of LTPA and PM_2.5_ with all-cause mortality among adults (aged 18 +), the following terms were used in full-text searches: (physical activity OR exercise OR sport OR leisure OR inactivity OR sedentary behavior) AND (mortality OR death OR fatal) AND (air quality OR air pollution OR particulate matter OR fine particle) AND (cohort OR longitudinal OR prospective) AND (Cox OR survival OR hazard OR risk OR relative risk). The full search strings for each database are provided in Additional file: Table S1.

The second strategy involved identifying additional studies from the articles excluded after full-text review, which might contain unpublished results on LTPA, PM_2.5_, and mortality in adults. One author (PK) contacted the principal investigators of these cohort studies to confirm that they met the following selection criteria and would participate in this study. Additional potentially relevant datasets were accessed based on the authors' awareness.

#### Selection process: inclusion and exclusion

Eligibility for including articles was based on the following criteria: (1) original journal articles published in English before 6 January 2025; (2) studies involving longitudinal design; (3) participants in the age range of 18 or above or the mean age in this range at baseline; (4) report on the independent association of LTPA with mortality after stratifying PM_2.5_ levels, or the joint association of LTPA and PM_2.5_ with mortality; and (5) reported effect estimates of relative risk (RR) or hazard ratios (HRs) with 95% confidence intervals (CIs) for mortality.

We applied exclusion criteria to articles that (1) focused on clinical populations such as patients with cardiovascular diseases, type 2 diabetes, or cancer etc.; (2) did not specifically report on LTPA, encompassing research focusing on overall physical activity or non-LTPA, including physical activities occurring in household, commuting/transportation, or work-related contexts; (3) measured LTPA but cannot determine the energy expenditure of LTPA (i.e., metabolic equivalent task-hours per week, MET-h/week); and (4) were conducted using duplicate cohort data.

Two reviewers (PK and LC) independently screened each record and article retrieved during the systematic review. This screening was guided by our predefined selection criteria. In instances where discrepancies arose between their assessments, PK and LC aimed to resolve these disagreements through a consensus-based approach. EndNote reference management software was used to manage records and assist in screening titles and abstracts.

#### Data extraction

One author (PK) extracted the following information from each eligible study: author(s), year of publication, country, study population (cohort, sample size/death, age at baseline, and proportion of female), follow-up period, LTPA, PM_2.5_, statistical models, covariates for adjustment, and the hazard ratio (HR) estimates with corresponding 95% CIs for the models. Another author (LC) cross-checked 100% of extracted data. Whenever there were differences in the evaluations made by reviewers PK and LC, they endeavored to address and resolve these discrepancies by reaching a consensus through discussion.

#### Risk of bias assessment

The research quality of the included studies was assessed using the Quality Assessment tool for Observational Cohort Studies and Cross-Sectional Studies developed by the US National Heart, Lung, and Blood Institute (NHLBI) [21, 22], including the checklist of the 14 items for assessing research quality of each study (https://www.nhlbi.nih.gov/health-topics/study-quality-assessment-tools) [23]. The research quality assessment process is detailed in Appendix.

#### Data harmonization and synthesis

The corresponding authors of each study were contacted and invited to participate in this harmonized meta-analysis. All LTPA data in the included studies were collected via self-reported questionnaires. They were asked to re-analyze their individual-level data based on a standardized protocol outlined in Additional file: Table S2 and then provide summary data. LTPA was calculated using MET-h/week and categorized into four levels based on the World Health Organization Physical Activity Guidelines: < 1 (least active), 1–7.5 (insufficiently active), 7.5–15 (recommended: at least 150–300 min of moderate-intensity physical activity), and 15 + MET-h per week (highly active: at least 300 + min of moderate-intensity physical activity). Except for one study that used quintiles [10], PM_2.5_ concentrations were categorized into quartiles based on the annual average during follow-up for stratification and to estimate the moderation effect of PM_2.5_ on the association between LTPA (exposure) and all-cause mortality (outcome). *P* values for testing the interaction effect between LTPA and PM_2.5_ were also estimated.

#### Data analysis

To explore the relationships of LTPA with all‐cause mortality, we used summary data extracted from all the prospective cohort studies. The maximally adjusted HRs from multivariable proportional hazards models were selected to minimize the potential confounding bias in each study.

#### Subgroup analysis

To aggregate findings across different studies, we conducted two random-effects subgroup analyses to assess the dose‐response relationships between 4-level LTPA and all-cause mortality. The first part examined the associations of LTPA with all-cause mortality within each study. Then, the second one was conducted at different levels of ambient PM_2.5_ exposure. The annual mean of PM_2.5_ (μg/m^3^) was classified into five strata, including < 10, 10–15, 15–25, 25–35, 35–50, which is based on the interim targets of the WHO Global Air Quality Guidelines [18].

#### Heterogeneity

Heterogeneity between studies was evaluated using the *Q* statistic (i.e., a measure of weighted squared deviations) and the *I*^2^ (i.e., the proportion of total variation explained by variation between studies). The* Q *value and degree of freedom were utilized to check whether the heterogeneity was statistically significant [24]. The *I*^2^ values of 25%, 50%, and 75% correspond to the low, medium, and high levels of heterogeneity [25].

#### Meta-regression

DerSimonian and Laird Random‐effects models were adopted in meta-regression. The Knapp-Hartung method was utilized in the meta-regression to adjust for the dispersion across studies [24]. To ensure adequate statistical power of meta-regression, we followed the recommendation of at least 10 effect estimates per covariate [26]. As PM_2.5_ was modeled as a study-level moderator. This approach differs from individual-level analyses, where interaction terms are required. The significance of PM_2.5_ in predicting effect sizes of LTPA–mortality associations serves as evidence of effect modification at the study level.

In meta-regression analyses, first, LTPA and PM_2.5_ were included in the meta‐regression model to examine whether the effects of LTPA on all‐cause mortality are moderated by the PM_2.5_ levels. (Model 1). Then, we included two potential moderators (mean age of samples and percentage of females) for adjustment (Model 2).

#### Sensitivity analysis

Based on Model 2, several sensitivity analyses were performed to assess the robustness of the findings after including more study‐level variables that contribute the heterogeneity across studies. Sample size at baseline, mean length of follow‐up, publication status (published vs. unpublished), analytical method (Cox regression vs. time-varying Cox regression), and scores of research quality were scrutinized in a univariate meta-regression model. The variables reaching the significance level (*p* < 0.05) were then included for sensitivity analyses.

#### Publication bias assessment

We examined publication bias using Egger’s test to assess funnel plot asymmetry [27]. A significant result in this test indicates an asymmetric funnel plot. This typically occurs when small studies with non-significant findings remain unpublished, skewing the overall effect size estimation. To further assess the potential impact of publication bias on our results, we employed Duval and Tweedie’s Trim and Fill tests, which extended beyond analyzing the studies included in our meta-analysis by imputing hypothetical missing studies to balance the funnel plot [28]. The funnel plot was also visually assessed for potential asymmetry.

#### Certainty of evidence

The Grading of Recommendations Assessment, Development and Evaluation (GRADE) system for grading evidence was employed to assess the certainty of evidence [29, 30]. Based on the GRADE system, it assesses the quality of a body of evidence as high, moderate, low, or very low [30].

A two-sided *p* value of < 0.05 was considered statistically significant. Unless otherwise indicated, all meta-analyses were conducted out using Comprehensive Meta-analysis Version 4.0 (Biostat) [31].

### Study two: individual-level pooled analysis of cohort studies

In this independent pooled analysis, individual-level data from three eligible cohorts were combined to evaluate the associations between LTPA and all-cause and cause-specific mortality across a broader spectrum of PM₂.₅ exposure levels. By integrating data at the participant level, this analysis achieved a substantially larger sample size, thereby increasing statistical power to detect small effect sizes, and expanded the exposure range beyond that of any single cohort. Ethical approval for this study was obtained from the Institutional Review Board of Taichung Veterans General Hospital in Taiwan (CE24585C).

#### Study population

The study sample comprised participants from three population-based prospective cohort studies participating in the international collaboration project. The project focuses on a systematic review and harmonized meta-analysis examining the associations between LTPA, PM_2.5_, and mortality. The inclusion of these three cohorts (UK Biobank, Taiwan Biobank, and MJ Cohort) was determined by the availability of individual-level data and the feasibility of data harmonization.

These datasets encompassed the UK Biobank (endorsed by the UK’s National Health Service, National Research Ethics Service: Ethics Committee reference No. 11/NW/0382) [32, 33], the Taiwan Biobank (authorized by the Ethics and Governance Council of Taiwan Biobank and the Department of Health and Welfare, Taiwan: Wei-Shu-I-Tzu No. 1010267471) [34], the Taiwan MJ Cohort (Ethical reviews approved by the Institutional Review Boards at the Taiwan National Health Research Institute) [35]. Because the complete cases (*n* = 713,120) represented 82.3% of the total sample (*n* = 869,038, with missing data exceeding 10%), the subsequent analyses were carried out after utilizing multiple imputation, employing an iterative Markov chain Monte Carlo approach with 20 imputations [36]. The details of the included cohort data were delineated in Additional file: Fig. S1.

##### Measures and data harmonization

#### Leisure-time physical activity

In each cohort study, participants’ engagement in LTPA was evaluated by collecting self-reported data on the frequency, type (or intensity), and duration of their activities. LTPA energy expenditure was categorized as follows: < 1 (inactive), 1–7.5 (insufficiently active), 7.5–15 (recommended: at least 150–300 min of moderate-intensity physical activity), and 15 + MET-h per week (highly active: at least 300 + minutes of moderate-intensity physical activity) [1, 37].

#### Ambient PM_2.5_: annual average over follow-up duration

1. UK Biobank: The estimates within the UK Biobank dataset were derived from diverse air pollution models, namely European Study of Cohorts for Air Pollution Effects (ESCAPE) (2010) and the EU-wide (2005–2007) model. Due to the incompatibility for combining and averaging these distinct models, solely the PM_2.5_ data from the year 2010 was employed. The PM_2.5_ estimates were generated for each participant's address utilizing a Land Use Regression model [38].
2. Taiwan Biobank and MJ Cohort: The PM_2.5_ monitoring data was acquired from the datasets of Environmental Protection Administration, Executive Yuan (https://data.epa.gov.tw). Within the MJ Cohort, yearly mean concentrations specific to the geocoded participants’ addresses were estimated for the period between 2005 and 2016 through 76 nationwide atmospheric monitoring sites in Taiwan [39]. In the Taiwan Biobank [40], comprehensive residential addresses of respondents were not made publicly available. Instead, annual average estimates were computed utilizing a consistent methodology based on the residential areas (townships/cities and districts) of participants throughout Taiwan. This computation spanned the years 2008 to 2020.

The annual mean PM_2.5_ concentrations for each participant during the follow-up period were categorized into five levels (< 10, 10–15, 15–25, 25–35, 35–50 μg/m^3^) [18, 41].

### Outcome: all-cause and cause-specific mortality

For the UK Biobank [42], the Taiwan Biobank [34], and the MJ Cohort [39], data on all-cause, cancer, and cardiovascular mortality over the follow-up were acquired through linkage with the respective government death registries. These registries provided information on individuals’ death status, causes of death, and dates of death. A detailed definition of ICD-10 codes for cause-specific mortality was provided in Additional file: Table S3.

### Covariates

In line with the guidelines provided by the recommendations of the bias assessment instrument for systematic reviews informing WHO global air quality guidelines [43], the following covariates at baseline were included: (i) socio-demographic characteristics: sex (male vs. female), age, educational level (low, medium, and high), household income (low, medium, and high); (ii) health-related behaviors: smoking (current smoker, former smoker, and never smoked), alcohol consumption (current drinker, former, and none/occasional); (iii) health status: weight status by body mass index (BMI) [44–46], and number of chronic diseases (including cancer, heart disease, stroke, hypertension, diabetes, liver disease, kidney disease, chronic pulmonary disease, and arthritis.); (iv) Cohort (study-level variable: UK Biobank, Taiwan Biobank, and MJ Cohort).

Given the inherent diversity in variable coding across the included studies, data harmonization was undertaken across the studies for the subsequent pooled data analysis (Additional file: Table S4).

### Statistical analysis

To examine the relationships between baseline LTPA and annual average PM_2.5_ levels during follow-up with all-cause and cause-specific mortality, we conducted the following analyses. First, we assessed the independent and joint associations of LTPA and PM_2.5_ on mortality, as well as evaluated consistency across subgroup populations, including sex, age groups (18–64 vs. 65 +), and the presence of cardiovascular diseases (yes vs. no). To evaluate the risk of different causes of death associated with the exposures of interest, we conducted a competing risk analysis using cause-specific Cox proportional hazards models [47].

Instead of performing separate stratified analyses within each PM₂.₅ exposure category—which may inadequately control for cohort-specific confounding—we examined the joint associations of LTPA and PM₂.₅ with mortality using a multivariable-adjusted model that accounted for cohort effects and employed a common reference group. This approach provides more consistent and interpretable estimates of the combined exposure effects across subgroups.

Before conducting the Cox regression analyses, the proportional hazards assumption in Cox regression was tested using time-dependent covariates [48]. Significant results (*p* < 0.001) indicate a potential violation of the assumption (Additional file: Table S5). However, in large sample sizes, even minor deviations can yield significant results. Therefore, we examined scaled Schoenfeld residual plots [49], where a flat line at zero on the y-axis suggests that the coefficient remains constant over time, confirming that the proportional hazards assumption holds (Additional file: Fig. S2).

Several sensitivity analyses were undertaken. First, individuals who died during the first 2 years of the mortality follow-up (*n* = 3213) were excluded to minimize the possibility of reverse causation bias using the imputed data. Second, to assess the impact of differences in LTPA measurements across cohorts, we implemented an alternative harmonized categorization of LTPA. Participants who did not engage in any LTPA served as the reference group, while those active during leisure time were categorized into tertiles based on their activity levels, measured in MET-hours per week. Third, we conducted a complete-case analysis restricted to participants without missing data (*n* = 713,120).

All analyses were conducted using SAS 9.4 software and a *p* value < 0.05 was considered statistically significant.

## Results

### Study one: systematic review and meta-analysis

**Fig. 1:**
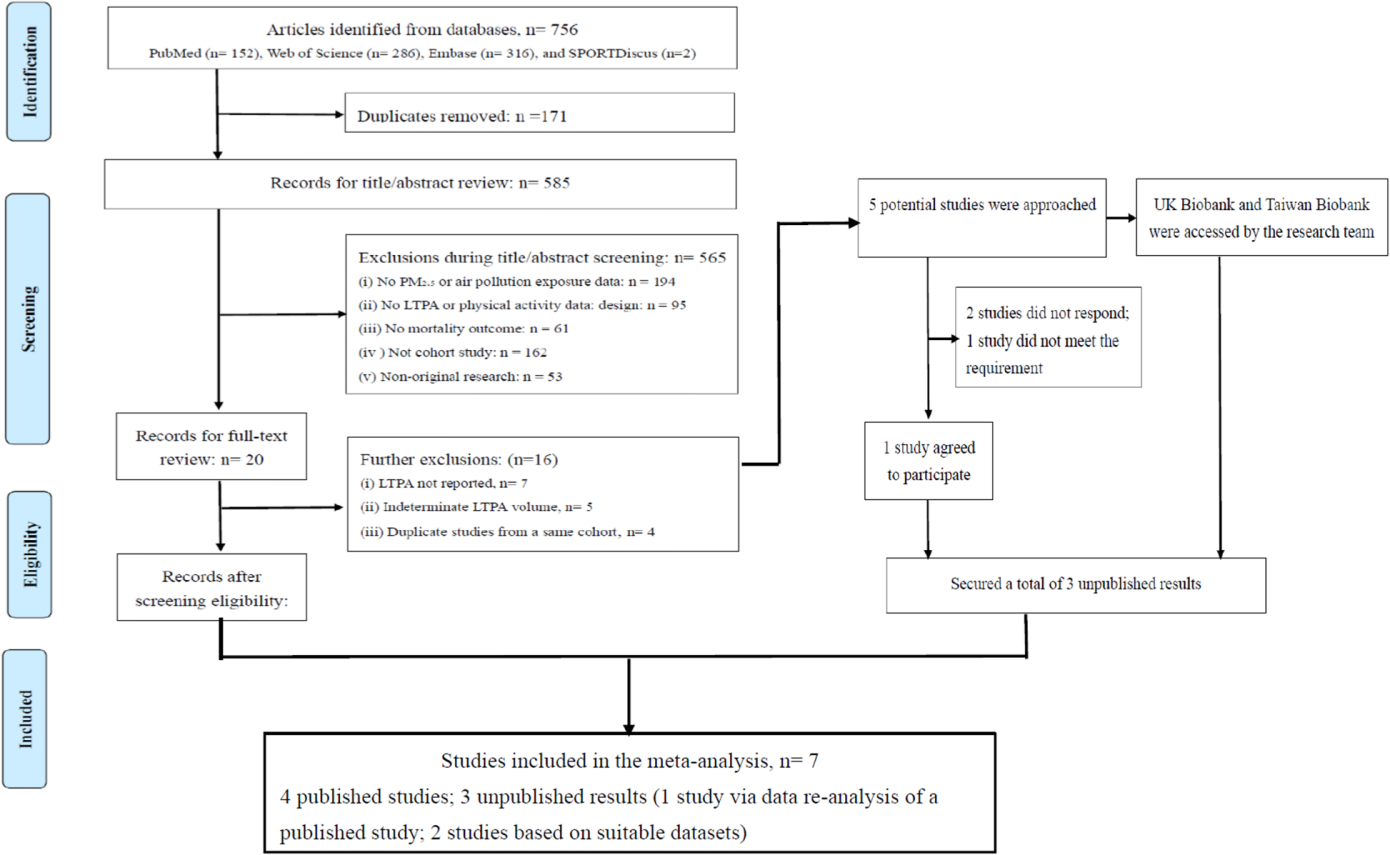
Flowchart of study selection in the systematic review and meta-analysis

The study selection process is shown in Fig. 1. We identified four studies through electronic data searches [10, 11, 14, 15]. Additionally, we identified five studies from the 16 excluded studies. These studies potentially had unpublished results on LTPA, PM_2.5_ exposure, and all-cause mortality in adults [50–54]. PK, one of the authors, reached out to the principal investigators of these cohort studies to confirm if they met our selection criteria. This process excluded one study based on the UK Biobank data [54], as we could access the UK Biobank data for data re-analysis to meet the research need. Among the four studies contacted, one agreed to participate [52], one did not meet the requirement [51], while the remaining two did not respond to our inquiries [50, 53]. Notably, the one participating studies were based on further analyses of LTPA using data from their previously published studies [52]. Additionally, we also accessed the Taiwan Biobank [34], which meet our research need. The 7 studies included 1.5 million participants (*n* = 1,515,094) with a median study follow-up time of 12.3 years and 115,196 reported deaths (Table 1). Detailed information of each study can be found in Additional file: Table S6. Notably, except for the studies in Taiwan (MJ Cohort and Taiwan Biobank), the tests for the interaction effect of LTPA and PM_2.5_ on mortality were not significant in the remaining 5 cohorts. The NHLBI Quality Assessment scores ranged between 10 and 11 out of a maximum possible score of 14. Overall, the studies were rated as fair (*n* = 3) to good (*n* = 4) quality (Additional file: Table S7).

**Table 1:** Characteristics of cohort studies included in the meta-analysis

Cohort	Author (year), Country	Sample, n/death (% of female)	Mean follow-up (years)	Annual average of PM_2.5_ (μg/m^3^)^b^/stratification	LTPA (MET-h/week)^b^
Published studies					
Nurses’ Health Study (NHS)	Elliott et al. (2020), US	104,990/9827 (100%)	20.0	Median = 13.4, IQR = 4.7/ Quintile	< 3.4, 3.4–10.2, 10.2–22.6, 22.6 +
Chinese Elderly Health Service (CEHS)	Sun et al. (2020), HK (SAR)	58,643/15,874 (65.7%)	10.3	Median = 35.3, IQR = 3.4/ Quartile	< 1, 1–7.5, 7.5–15, 15 +
MJ Cohort (MJ)	Guo et al. (2021), Taiwan (R.O.C.)	384,130/12,375 (51.3%)	13.4	Median = 23.9, IQR = 6.2/ Quartile	< 1, 1–7.5, 7.5–15, 15 +
US National Health Interview Survey (NHIS)	Coleman et al. (2022), USA	403,748/39,528 (54.7%)	7.0	Median = 10.6, IQR = 3.2/ Quartile	< 1, 1–7.5, 7.5–15, 15 +
Unpublished results					
Danish Diet, Cancer and Health Cohort (DDCHC)	Hvidtfeldt & Raaschou-Nielsen (2023), Denmark	49,560/10,190 (53.1%)	18.1	Median = 14.5, IQR = 1.2/ Quartile	< 1, 1–7.5, 7.5–15, 15 +
UK Biobank (UKB)	Xia (2024), UK	379,663/26,206 (52.5%)	12.3	Median = 9.9, IQR = 1.3/ Quartile	< 1, 1–7.5, 7.5–15, 15 +
Taiwan Biobank (TWB)	Ku (2025), Taiwan (R.O.C.)	134,360/1196 (64.0%)	5.2	Median = 18.8, IQR = 7.7/ Quartile	< 1, 1–7.5, 7.5–15, 15 +
**Total n of cohort studies** = **7**		**Total n/death** = **1,515,094/115,196**			

*HK* (SAR) Hong Kong (Special Administrative Region of China), *R*.*O*.*C*. Republic of China, *IQR* interquartile range, *LTPA* leisure-time physical activity, *MET*-*h* metabolic equivalent hours, *NS* non-significant, *PM*_*2*.5_ particulate matter 2.5

^a^Age at baseline

^b^Details on the assessment of PM_2.5_, LTPA, and covariates and data harmonization can be found in Additional file: Table S2; annual average of PM_2.5_ in the pooled analyses categorized into 5 levels: < 10, 10–15, 15–25, 25–35, and 35–50 with the upper bound exclusive

#### Subgroup analyses

**Fig. 2:**
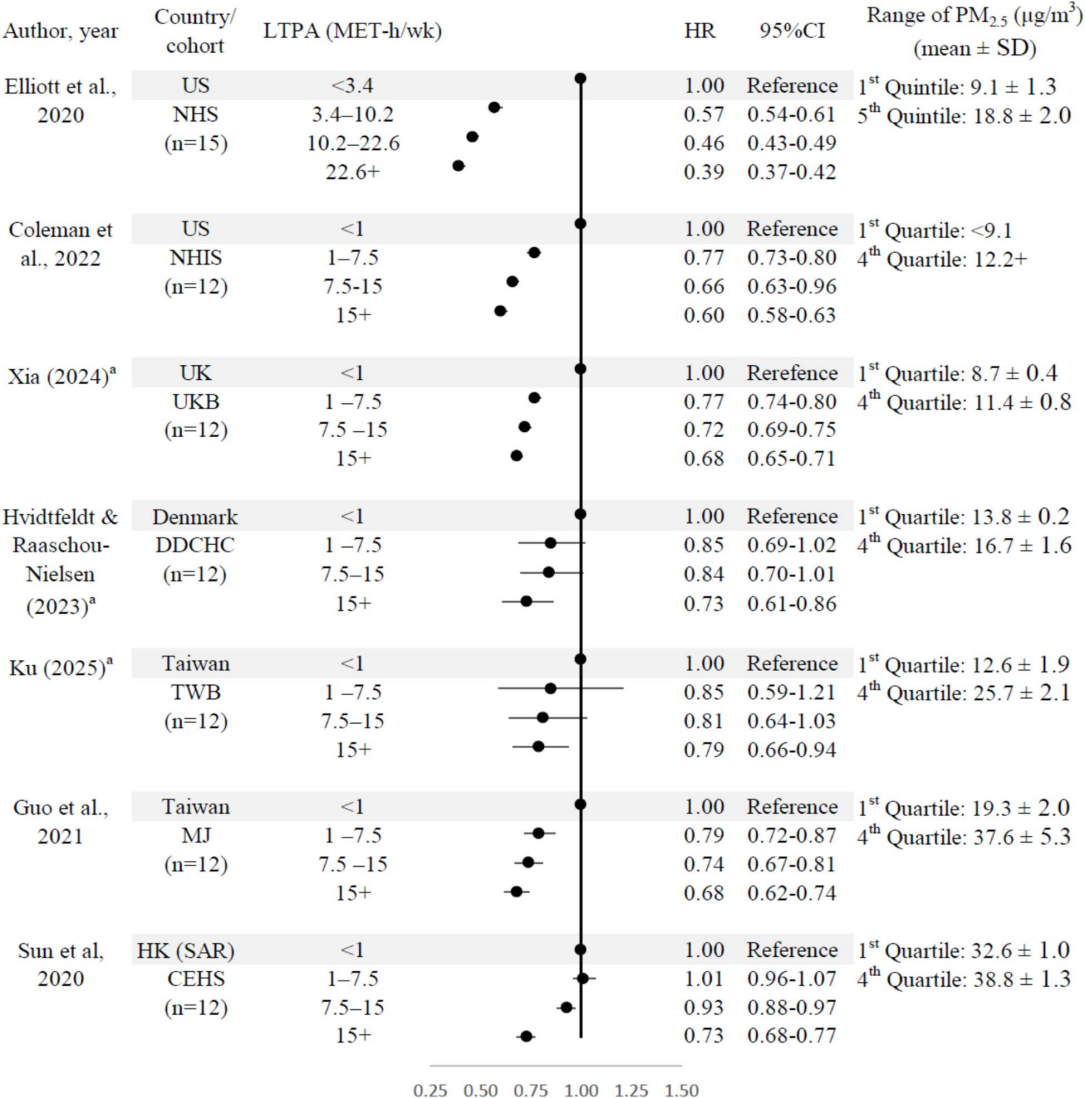
Subgroup analyses of meta-analysis by cohorts A unpublished results. *PM*_*2*.5_ fine particulate matter 2.5, *LTPA* leisure-time physical activity, *HR* hazard ratio, ‘*n*’ in the brackets represents the number of effect estimates in each study. LTPA was categorized into four levels in each study, while PM_2.5_ concentrations were categorized into quartiles, except for one study that used quintiles for PM_2.5_ [6]. Therefore, six studies yielded 72 effect estimates (6 studies * 3 levels * 4 categories), and one study (Elliot et al., 2020) yielded 15 estimates (1 study * 3 levels * 5 categories). Total number of effect estimates = 87. The data shown in the figures were derived from individual cohorts, and details on the covariates available and adjusted for in each cohort were provided in Additional file: Table S6. The category ranges of LTPA were defined as lower bound inclusive and upper bound exclusive, except for the highest category (15 + MET-h/week), which was open-ended

**Fig. 3:**
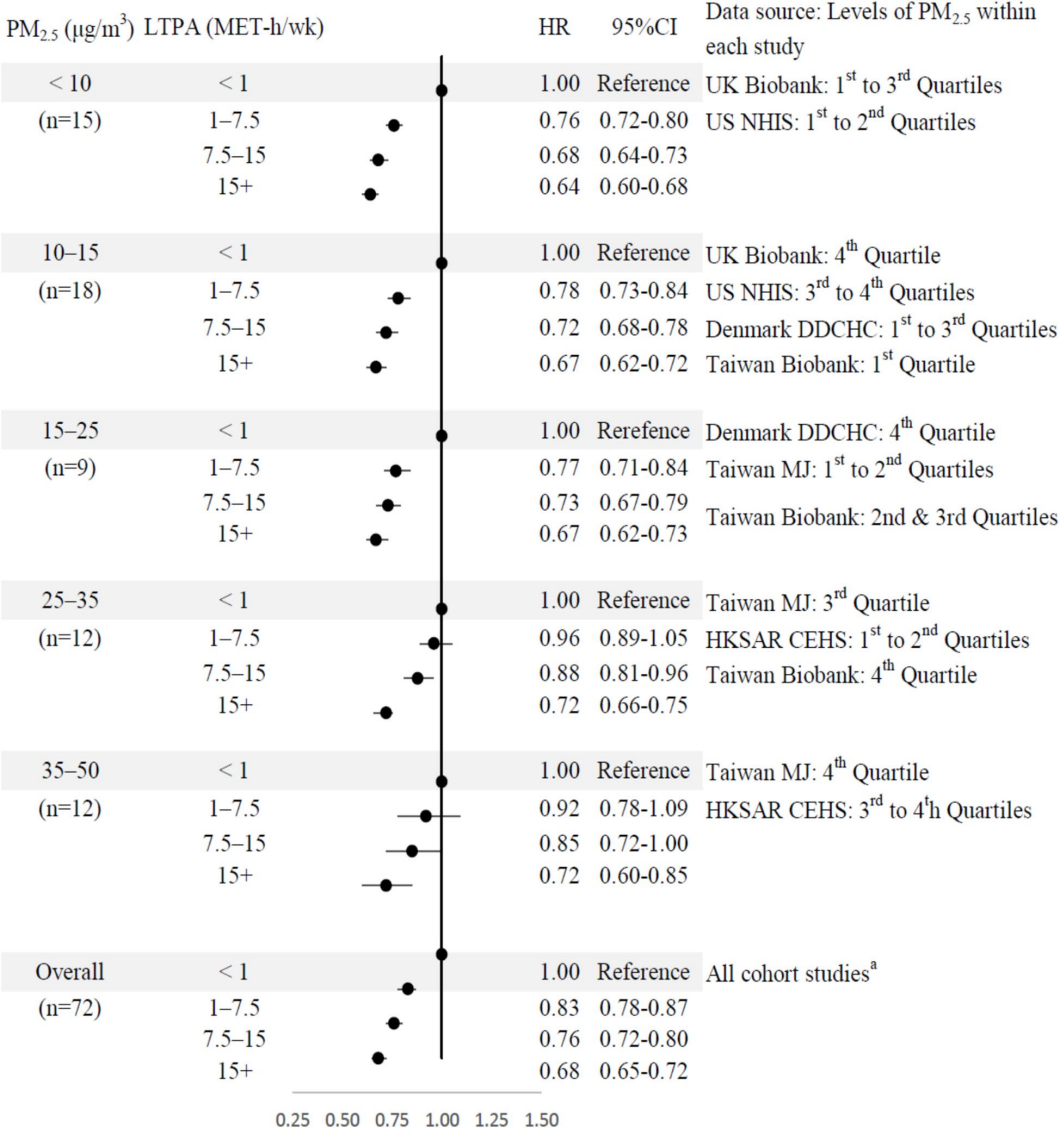
Subgroup analyses of meta-analysis by PM_2.5_ levels *NHS* Nurses’ Health Study; *NHIS* National Health Interview Survey; *UKB* UK Biobank; *DDCHC* Danish Diet, Cancer and Health Cohort; *TWB* Taiwan Biobank; *MJ* MJ Cohort Study; *HKSAR* Hong Kong Special Administrative Region; *CEHS* Chinese Elderly Health Service. The data shown in the figures were derived from individual cohorts, and details on the covariates available and adjusted for in each cohort were provided in Additional file: Table S6. All category ranges of LTPA and PM_2.5_ were defined as lower bound inclusive and upper bound exclusive, except for the highest category (15+ MET-h/week), which was open-ended

The first random‐effects subgroup analysis demonstrated that greater engagement in LTPA is progressively associated with lower all‐cause mortality within each study. However, the benefits of LTPA on all-cause mortality appeared to be attenuated in regions with higher levels of PM_2.5_ compared to those with lower PM_2.5_ exposure (Fig. 2). The second subgroup analysis assessed the relationships of LTPA with all-cause mortality at different levels of ambient PM_2.5_ exposures (Fig. 3). The subgroup analysis indicated a dose‐response relationship between LTPA and mortality (overall, *n* = 72). Engaging in LTPA at a level of 7.5–15 MET-h/week (equivalent to 150–300 min of moderate-intensity physical activity per week) was associated with a reduction of approximately 30% in mortality risk at PM_2.5_ below 25 μg/m^3^. However, the benefits of LTPA decreased to around 12% to 15% reduction in mortality risk at PM_2.5_ 25 + μg/m^3^.

#### Meta-regression

In Model 1 of the meta-regression, engaging in more LTPA was associated with lower mortality (coefficient = − 0.15, *p* < 0.001), and the protective association was significantly moderated by PM_2.5_ levels (*p* < 0.001) (Additional file: Table S8). However, the protective effect of LTPA against mortality did not differ significantly across PM_2.5_ categories below 25 μg/m^3^, although a borderline association was observed in the 15–25 μg/m^3^ group in Models 2 and 3. In contrast, at higher PM_2.5_ levels (25 + μg/m^3^), the regression coefficients were significantly greater than zero, indicating that as air pollution concentrations increase, the protective association of LTPA was attenuated and mortality risk increased.

Compared with the lowest level of PM_2.5_ (< 10 μg/m^3^), the protective effect of LTPA was not significantly altered at the range of PM_2.5_ 10–25 μg/m^3^. In contrast, associations of LTPA with all-cause mortality were significantly weakened when PM_2.5_ was greater than 25 μg/m^3^. After including several study-level variables (i.e., ‘mean age of samples’, ‘proportion of females’), the Model 2 revealed similar patterns.

#### Sensitivity analyses

We further included two study-level variables (i.e., ‘mean length of follow-up’, and ‘publication status’), which were significantly associated with mortality risk in a univariate meta-regression model. Model 3 and Model 4 showed similar results (Additional file: Table S8). Overall, more LTPA was consistently associated with lower mortality (*p* < 0.001). However, the protective associations between LTPA and mortality were significantly moderated by PM_2.5_ levels (*p* < 0.001), particularly at concentrations of PM_2.5_ 25 + μg/m^3^.

#### Evaluation of publication bias

The funnel plot was asymmetric, suggesting possible bias (Additional file: Fig. S3). However, Egger’s test indicated no significant evidence of publication bias (two-tailed *p* value = 0.30). Similarly, under the random-effects model, the point estimate for HR for the combined studies (0.69, 95% CI = 0.66 to 0.72) was slightly higher than the adjusted estimate after imputing several studies (0.64, 95% CI = 0.61 to 0.67) in the Trim and Fill adjustment.

#### Assessment of certainty of evidence

Overall, we judged the certainty of evidence to be moderate. The descriptions associated with this analysis and the according explanations of the rationale behind the judgements can be seen in Additional file: Table S9.

### Study two: individual-level pooled analysis of cohort studies

#### Study characteristics

Study Two reports the findings from an independent pooled analysis of three eligible cohorts. The flowchart of sample selection process was shown in Additional file: Fig. S1. An aggregate of 869,038 adults aged 18 or older participated, with a cumulative count of 45,080 reported deaths. The mean follow-up duration was 11.29 (SD = 3.52) years. The pooled analysis integrated three distinct cohort studies situated across regions characterized by varying levels of PM_2.5_ (Additional file: Fig. S4). The relationships between baseline characteristics and all-cause and cause-specific mortality are outlined in Table 2. The descriptive statistics for LTPA engagement at baseline are presented in Additional file: Table S10.

**Table 2:** Baseline sample characteristics of the pooled individual participant data (*n* = 869,038)

Variables	*n*	All-cause mortality		Cancer mortality		Cardiovascular mortality	
		%	*P* value for χ^2^	%	*P* value for χ^2^	%	*P* value for χ^2^
Socio-demographic							
Sex			< 0.001		< 0.001		< 0.001
Male	389,337	7.0		3.1		1.6	
Female	479,701	3.9		2.2		0.5	
Age			< 0.001		< 0.001		< 0.001
65 +	118,129	15.7		7.0		3.3	
45–65	481,097	5.2		2.7		0.9	
18–45	269,812	0.8		0.4		0.1	
Educational level^a^			< 0.001		< 0.001		< 0.001
Low	261,385	8.9		4.2		1.8	
Medium	352,073	4.9		2.5		0.9	
High	255,580	2.0		1.0		0.4	
Household annual income^a^			< 0.001		< 0.001		< 0.001
Low	225,133	8.1		3.5		1.7	
Medium	373,973	4.9		2.6		0.9	
High	269,932	2.2		1.3		0.4	
Cohort			< 0.001		< 0.001		< 0.001
UK Biobank	501,088	7.6		3.8		1.5	
Taiwan Biobank	135,261	0.9		0.4		0.2	
MJ Cohort	232,689	2.9		1.2		0.5	
Health-related behaviors							
LTPA (MET-h/week)			< 0.001		< 0.001		< 0.001
< 1	229,890	6.4		2.8		1.4	
1–7.5	313,974	4.4		2.3		0.8	
7.5–15	135,168	5.3		2.8		1.0	
15 +	190,006	5.0		2.6		0.9	
Smoking			< 0.001		< 0.001		< 0.001
Former	201,851	8.3		4.1		1.6	
Current	111,571	8.4		3.9		1.8	
Never	555,616	3.5		1.8		0.6	
Alcohol consumption			< 0.001		< 0.001		< 0.001
Former	27,678	11.2		4.5		2.3	
Current	501,237	7.0		3.6		1.3	
None/occasional	340,123	2.2		1.0		0.4	
Health Status							
Number of chronic diseases			< 0.001		< 0.001		< 0.001
2 +	84,554	15.7		6.4		4.0	
1	218,864	7.3		3.8		1.3	
0	565,620	2.9		1.6		0.4	
Body mass index^b^			< 0.001		< 0.001		< 0.001
Obese	144,662	8.6		4.0		2.0	
Overweight	306,328	5.9		3.0		1.1	
Normal	389,884	3.6		1.8		0.6	
Underweight	28,164	2.8		1.1		0.4	

The category ranges of age, LTPA, and body mass index were defined as lower bound inclusive and upper bound exclusive, except for the highest categories, which was open-ended

*LTPA* leisure-time physical activity, *MET*-*h* metabolic equivalent hours

^a^Details on the original categories and data harmonization can be found in Additional file: Table S4

^b^Cut-offs of body mass index (underweight, normal, overweight, and obese) were based on country-specific recommendations: UK (< 18.5, 18.5–23.0, 23.0–25.0, 25.0 +), and Taiwan (< 18.5, 18.5–24.0, 24.0–27.0, 27.0 +)

#### Independent associations of LTPA and PM_2.5_ with mortality

After multivariable adjustment, both LTPA and PM_2.5_ were significantly associated with all-cause mortality. Furthermore, we found dose–response relationships of LTPA and PM_2.5_ with all-cause mortality (*p* values for linear trend < 0.001). Higher levels of LTPA and lower levels of PM_2.5_ were associated with a reduced risk of death (Additional file: Table S11).

#### Joint associations of LTPA and PM_2.5_ with mortality

**Fig. 4:**
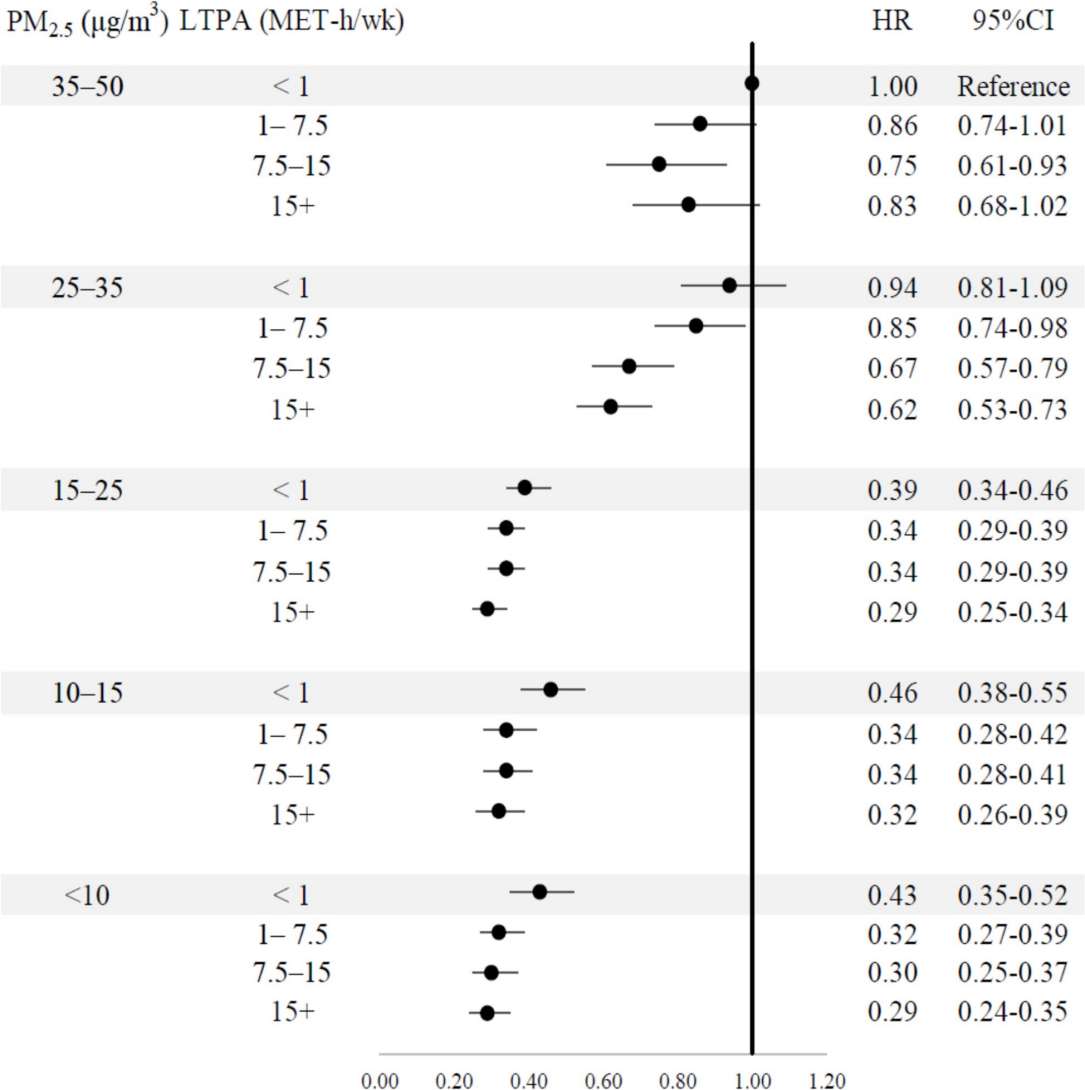
Joint associations of leisure-time physical activity and ambient PM_2.5_ with all-cause mortality in the pooled individual participant data (*n* = 869,038) *CI* confidence interval, *HR* hazard ratio, *LTPA* leisure-time physical activity, *MET*-*h* metabolic equivalent hours, *PM*_*2*__*.5*_ particulate matter 2.5. Covariates: sex, age, educational levels, household income, smoking, alcohol consumption, body mass index, number of chronic diseases, and cohort. The category ranges of PM_2.5_ and LTPA were defined as lower bound inclusive and upper bound exclusive, except for the highest category (i.e., 15+ MET-h/wk), which was open-ended

We assigned a single reference category comprising participants exposed to low LTPA group (LTPA: < 1 MET-h/week) and high air pollution (PM_2.5_: 35–50 μg/m^3^) (Fig. 4). Individuals meeting the recommended LTPA level (7.5–15 MET-h/week, equivalent to 150–300 min of moderate-intensity physical activity per week) had a lower risk of all-cause mortality than those in the highest-risk group (< 1 MET-h/week and PM_2.5_: 35–50 μg/m^3^). Hazard ratios (HRs) varied by PM_2.5_ exposure, with lower HRs indicating a greater protective effect. For example, those exposed to PM_2.5_ of 35–50 μg/m^3^ (HR = 0.75, 95% CI: 0.61–0.93), followed by 25–35 μg/m^3^ (HR = 0.67, 95% CI: 0.57–0.79), 15–25 μg/m^3^ (HR = 0.34, 95% CI: 0.29–0.39), 10–15 μg/m^3^ (HR = 0.34, 95% CI: 0.28–0.41), and < 10 μg/m^3^ (HR = 0.29, 95% CI: 0.24–0.35). The protective effect of LTPA diminished notably at PM_2.5_ levels of 25 + compared to levels under 25.

**Fig. 5:**
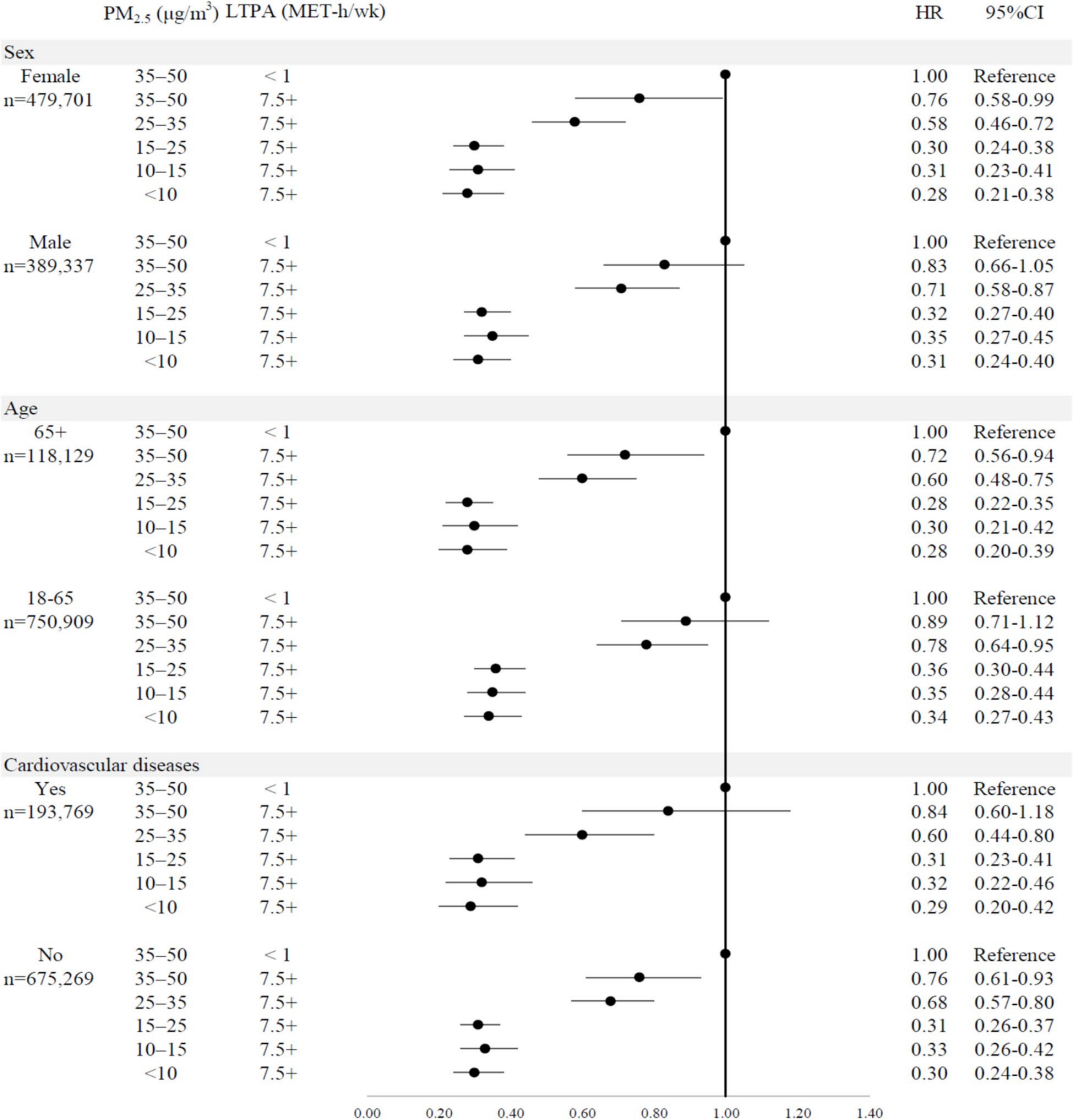
Joint associations of leisure-time physical activity and PM_2.5_ with all-cause mortality across subgroup populations in the pooled individual participant data (total *n* = 869,038) *PM*_*2*__*.5*_ particulate matter 2.5, *LTPA* leisure-time physical activity, *HR* hazard ratio. Covariates: in all models: sex, age, educational levels, household income, smoking, alcohol consumption, body mass index, number of chronic diseases, and cohort. The reference group was defined as individuals exposed to the highest risk levels of PM_2.5_ at a concentration of 35–50 μg/m^3^ and engaging in the least active group (< 1 MET-h/wk). To simplify the presentation and facilitate comparison across subgroups, only the results for participants meeting the WHO-recommended level (7.5+ MET-h/week) were shown, while lower activity groups were not included. The category ranges of PM_2.5_ and LTPA were defined as lower bound inclusive and upper bound exclusive, except for the highest category (i.e., 7.5+ MET-h/wk), which was open-ended

The joint-association analysis was stratified by sex (females vs. males), age group (65 + vs. 18–64), and the presence of cardiovascular diseases (yes vs. no), as shown in Fig. 5. For individuals meeting or exceeding the recommended level of LTPA (7.5 + MET-h/week, equivalent to 150 + min of moderate-intensity physical activity per week), patterns similar to those in the overall population (Fig. 4) were observed. Across all subgroups, the attenuation effect began at a PM_2.5_ concentration of 25 + μg/m^3^. At PM_2.5_ levels of 35–50 μg/m^3^, protective associations were further weakened (Fig. 5).

The joint associations with cancer and cardiovascular mortality across subgroup populations are shown in Table 3. Similar to the patterns for all-cause mortality, attenuation of the protective effects of LTPA emerged at PM_2.5_ levels of 25 + μg/m^3^ across all outcomes. At 35–50 μg/m^3^, the associations became non-significant in most subgroups, particularly for cancer mortality, where point estimates consistently approached or exceeded 1.0. Several sensitivity analyses were conducted to address potential reverse causality, differences in LTPA measurement across cohorts, and missing data. The results were consistent with the main analyses, further supporting the robustness of the findings (Additional file: Table S12, S13, and S14).

**Table 3:** Joint associations of leisure-time physical activity and PM_2.5_ with all-cause and specific-cause mortality across subgroup populations (total *n* = 869,038)

Population	PM_2.5_ (μg/m^3^)	LTPA (MET-h/week)	All-cause mortality^a^		Cancer mortality^a,b^		Cardiovascular mortality^a,b^	
			Death	aHR (95% CI)	Death	aHR (95% CI)	Death	aHR (95% CI)
Total								
*n* = 869,038	35–50	< 1	45,804	Ref	22,465	Ref	8771	Ref
	35–50	7.5 +		**0.79 (0.67, 0.94)**		1.09 (0.83, 1.42)		**0.61 (0.40, 0.92)**
	25–35	7.5 +		**0.65 (0.56, 0.75)**		0.80 (0.63, 1.02)		**0.50 (0.36, 0.71)**
	15–25	7.5 +		**0.31 (0.27, 0.36)**		**0.38 (0.30, 0.48)**		**0.30 (0.22, 0.42)**
	10–15	7.5 +		**0.33 (0.27, 0.40)**		**0.38 (0.28, 0.51)**		**0.37 (0.24, 0.57)**
	< 10	7.5 +		**0.30 (0.25, 0.36)**		**0.36 (0.27, 0.49)**		**0.32 (0.21, 0.50)**
Subgroup								
Sex								
Female	35–50	< 1	18,704	Ref	10,331	Ref	2638	Ref
(*n* = 479,701)	35–50	7.5 +		**0.76 (0.58, 0.99)**		1.13 (0.77, 1.68)		0.68 (0.35, 1.32)
	25–35	7.5 +		**0.58 (0.46, 0.72)**		**0.67 (0.47, 0.94)**		**0.44 (0.25, 0.77)**
	15–25	7.5 +		**0.30 (0.24, 0.38)**		**0.36 (0.25, 0.50)**		**0.30 (0.18, 0.51)**
	10–15	7.5 +		**0.31 (0.23, 0.41)**		**0.32 (0.21, 0.50)**		**0.47 (0.23, 0.95)**
	< 10	7.5 +		**0.28 (0.21, 0.38)**		**0.31 (0.20, 0.48)**		**0.42 (0.21, 0.85)**
Male	35–50	< 1	27,100	Ref	12,134	Ref	6133	Ref
(*n* = 38,9337)	35–50	7.5 +		0.83 (0.66, 1.05)		1.07 (0.73, 1.57)		0.59 (0.35, 1.01)
	25–35	7.5 +		**0.71 (0.58, 0.87)**		0.92 (0.66, 1.29)		**0.56 (0.37, 0.87)**
	15–25	7.5 +		**0.32 (0.27, 0.40)**		**0.40 (0.29, 0.56)**		**0.32 (0.21, 0.48)**
	10–15	7.5 +		**0.35 (0.27, 0.45)**		**0.43 (0.28, 0.65)**		**0.35 (0.20, 0.60)**
	< 10	7.5 +		**0.31 (0.24, 0.40)**		**0.40 (0.27, 0.62)**		**0.30 (0.18, 0.52)**
Age								
65 +	35–50	< 1	18,586	Ref	8252	Ref	3857	Ref
(*n* = 118,129)	35–50	7.5 +		**0.72 (0.56, 0.94)**		1.14 (0.73, 1.79)		0.59 (0.33, 1.06)
	25–35	7.5 +		**0.60 (0.48, 0.75)**		0.71 (0.48, 1.07)		**0.51 (0.31, 0.84)**
	15–25	7.5 +		**0.28 (0.22, 0.35)**		**0.32 (0.22, 0.48)**		**0.30 (0.18, 0.48)**
	10–15	7.5 +		**0.30 (0.21, 0.42)**		**0.35 (0.20, 0.59)**		**0.32 (0.16, 0.68)**
	< 10	7.5 +		**0.28 (0.20, 0.39)**		**0.34 (0.20, 0.57)**		**0.30 (0.14, 0.62)**
18–65	35–50	< 1	27,218	Ref	14,213	Ref	4914	Ref
(*n* = 750,909)	35–50	7.5 +		0.89 (0.71, 1.12)		1.15 (0.82, 1.63)		0.61 (0.34, 1.11)
	25–35	7.5 +		**0.78 (0.64, 0.95)**		1.01 (0.75, 1.36)		**0.57 (0.35, 0.90)**
	15–25	7.5 +		**0.36 (0.30, 0.44)**		**0.45 (0.33, 0.60)**		**0.32 (0.20, 0.51)**
	10–15	7.5 +		**0.35 (0.28, 0.44)**		**0.41 (0.28, 0.59)**		**0.39 (0.23, 0.68)**
	< 10	7.5 +		**0.34 (0.27, 0.43)**		**0.41 (0.28, 0.59)**		**0.36 (0.21, 0.61)**
CVDs								
Yes	35–50	< 1	21,026	Ref	8766	Ref	5341	Ref
(*n* = 193,769)	35–50	7.5 +		0.84 (0.60, 1.18)		1.16 (0.66, 2.02)		0.71 (0.38, 1.33)
	25–35	7.5 +		**0.60 (0.44, 0.80)**		0.65 (0.39, 1.08)		**0.39 (0.22, 0.68)**
	15–25	7.5 +		**0.31 (0.23, 0.41)**		**0.33 (0.20, 0.55)**		**0.26 (0.15, 0.45)**
	10–15	7.5 +		**0.32 (0.22, 0.46)**		**0.30 (0.17, 0.54)**		**0.34 (0.18, 0.65)**
	< 10	7.5 +		**0.29 (0.20, 0.42)**		**0.28 (0.16, 0.51)**		**0.29 (0.15, 0.56)**
No	35–50	< 1	24,778	Ref	13,699	Ref	3430	Ref
(*n* = 675,269)	35–50	7.5 +		**0.76 (0.61, 0.93)**		1.04 (0.76, 1.43)		**0.46 (0.26, 0.83)**
	25–35	7.5 +		**0.68 (0.57, 0.80)**		0.87 (0.66, 1.15)		**0.63 (0.41, 0.97)**
	15–25	7.5 +		**0.31 (0.26, 0.37)**		**0.40 (0.31, 0.53)**		**0.33 (0.22, 0.51)**
	10–15	7.5 +		**0.33 (0.26, 0.42)**		**0.43 (0.30, 0.61)**		**0.38 (0.21, 0.69)**
	< 10	7.5 +		**0.30 (0.24, 0.38)**		**0.40 (0.28, 0.58)**		**0.33 (0.18, 0.61)**

*aHR* adjusted hazard ratio, *CI* confidence interval, *CVDs* cardiovascular diseases, *LTPA* leisure-time physical activity, *MET*-*h* metabolic equivalent hours, *PM*_*2*.5_ particulate matter 2.5

Bold values indicate statistical significance

The reference group was defined as individuals exposed to the highest risk levels of PM_2.5_ at a concentration of 35–50 μg/m^3^ and engaging in the least active group (< 1 MET-h/wk). To simplify the presentation and facilitate comparison across subgroups, only the results for participants meeting the WHO-recommended level (7.5 + MET-h/week) were shown, while lower activity groups were not included

The category ranges of PM_2.5_ and LTPA were defined as lower bound inclusive and upper bound exclusive, except for the highest category (i.e., 7.5 + MET-h/wk), which was open-ended

^a^Adjusted for sex, age, educational levels, household income, smoking, alcohol consumption, body mass index, number of chronic diseases, and cohort

^b^Using cause-specific hazard models for competing risks of other mortality causes

## Discussion

This study represents a pioneering effort to evaluate whether the protective association of LTPA with all-cause, cardiovascular, and cancer mortality is attenuated by long-term exposure to ambient PM_2.5_ concentrations and to identify the threshold at which such attenuation emerges. By integrating evidence from two independent studies—a systematic review and meta-analysis, and an individual-level pooled analysis of cohort studies—this study examined the interplay among LTPA, air pollution, and mortality across PM_2.5_ levels from low (< 10 μg/m^3^) to very high (35–50 μg/m^3^). Key findings indicate that LTPA remained beneficial for all-cause, cardiovascular, and cancer mortality even under high PM₂.₅ levels. However, its protective effects were attenuated at 25 + μg/m^3^ for all outcomes and became minimal or non-significant at 35–50 μg/m^3^, particularly in relation to cancer mortality. These patterns are consistent across sex, age groups, and individuals with cardiovascular disease, which was further supported by sensitivity analyses.

Unlike prior investigations conducted within single countries with participants exposed to narrower PM_2.5_ ranges [10, 11, 14, 15, 39]. The meta-analysis provided robust summary estimates across multiple cohorts. Separately, the pooled analysis increased the likelihood of detecting small effect sizes by leveraging individual-level data and a wider exposure range. This supports the hypothesis that limited variability in PM_2.5_ levels within individual studies may have constrained their ability to address this question effectively.

This study found that the protective associations of LTPA with all-cause and cause-specific mortality were notably attenuated at PM_2.5_ levels of 25 + μg/m^3^. The observed association patterns were consistent across diverse population segments, suggesting that the relationships between PM_2.5_, LTPA, and mortality are robust. This strengthens the validity of our findings, indicating that the effects are not confined to specific demographic groups.

Moreover, prolonged exposure of PM_2.5_ increases lung and systemic inflammation, endothelial dysfunction, and arterial stiffness—key risk factors for cardiovascular diseases and mortality [6]. These factors may partly account for why protective association weakened at high PM_2.5_ concentrations (35 + μg/m^3^), particularly for cancer mortality and among people with cardiovascular diseases. These findings underscore the complex interactions between air pollution, physical activity, and mortality, with varying effects across different causes of death and population subgroups.

Although the confidence intervals at high PM₂.₅ concentrations (35–50 μg/m^3^) were wide and statistically non-significant, the point estimates for cancer mortality consistently approached or exceeded 1.0 across all subgroups. This pattern may indicate a diminished protective effect of LTPA under conditions of high ambient air pollution. Furthermore, due to the limited exposure range in our data, it remains uncertain whether the protective effect weakens further or ultimately reverses at PM_2.5_ concentrations exceeding 50 μg/m^3^.

Our findings emphasize that engaging in LTPA in regions with better air quality could enhance its health benefits. Unfortunately, global exposure estimates highlight that poor air quality poses significant health risks to a vast majority of the global population. Approximately 46% of the global population lives in areas where the annual mean PM₂.₅ concentration exceeds 25 μg/m^3^, and around 36% are exposed to levels exceeding 35 μg/m^3^ [55]. This underscores the need for international efforts to reduce fine particle emissions from transportation, industry, and other sources.

In the present study, we focus on the joint association of LTPA and PM₂.₅ with mortality. This is because the substantial differences in PM₂.₅ distributions across cohorts mean that stratified or interaction models necessarily involve different reference groups, which limits comparability across studies. For completeness, we also tested the statistical interaction between LTPA and PM₂.₅ (Additional file: Table S15). Both the multiplicative interaction and the additive interaction in some subgroups reached statistical significance, indicating an antagonistic association between them—that is, the protective effect of LTPA was weakened at higher PM₂.₅ levels. Nevertheless, given these limitations, the joint association framework provides a more consistent and interpretable approach for examining the combined effects across cohorts. Collectively, this convergence across meta-regression and individual participant data (IPD) cohort analysis strengthens the validity of our findings, indicating that the observed moderation by PM₂.₅ is robust to both study-level and individual-level modeling frameworks.

From a clinical and public health perspective, these findings suggest that LTPA remains beneficial even at PM₂.₅ levels above 25 μg/m^3^, highlighting the importance of promoting physical activity alongside air pollution mitigation. In areas with annual PM₂.₅ levels exceeding 25 μg/m^3^, policies could focus on encouraging LTPA while implementing protective measures, such as incorporating air quality considerations into exercise guidelines, recommending reduced-intensity or indoor activities during high-pollution days, and expanding access to low-pollution green spaces to help maximize the health benefits of LTPA.

This study is the first to integrate a systematic review, meta-analysis, and individual-level pooled analysis of cohort studies to examine the influence of PM_2.5_ on the relationship between LTPA and mortality risks. Its strengths include a large sample size, diverse PM_2.5_ concentrations, and data from multiple countries, enhancing the detection of attenuation effects, the identification of thresholds, and generalizability. Furthermore, it examines both all-cause and cause-specific mortality, exploring potential variations across mortality causes and subgroup populations.

This study has several limitations. The limited number of cohort studies, most of which were conducted in high-income countries, may reduce the generalizability of the findings to low- and middle-income regions, where PM₂.₅ levels often exceed 50 μg/m^3^ but monitoring data are sparse. Therefore, the applicability of our findings to regions with persistently high PM_2.5_ concentrations (> 50 µg/m^3^) remains uncertain and warrants further investigation. Moreover, although we applied harmonized analytic strategies and adjusted for cohort effects, the pooled analysis was based on cohorts from only two countries (UK and Taiwan). The imbalance in regional representation across PM₂.₅ categories may therefore have introduced bias (Additional file: Table S16), and this limitation should be considered when interpreting the findings. Variability in PM₂.₅ assessment methods and the lack of indoor air quality data (e.g., household or occupation exposure) further constrain the interpretation of the results. Additionally, potential residual confounding factors such as dietary patterns were not assessed in the included studies, and these unmeasured factors may have contributed to the observed associations and should be considered in future research. Finally, LTPA was assessed inconsistently across studies and relied on self-reported data. Such self-reporting may introduce misclassification bias, which could either attenuate or exaggerate the true association between LTPA and exposure outcomes. However, sensitivity analyses addressing differences in LTPA measurement yielded consistent results. Despite these limitations, prior research indicates that over 85% of participants in the pooled cohorts engaged in outdoor LTPA [14, 15, 56], reinforcing the relevance of outdoor activities in this study.

## Conclusions

LTPA appeared to remain beneficial for all-cause, cardiovascular, and cancer mortality even at relatively high PM₂.₅ levels, with the benefits tending to be greater under cleaner air conditions. However, its protective effects seemed to be attenuated at concentrations ≥ 25 μg/m^3^ for all outcomes and became less evident at 35–50 μg/m^3^, particularly for cancer mortality. These findings suggest the importance of considering air quality in physical activity and public health guidelines. Given these limitations, our findings should be interpreted with caution, and further studies across more diverse exposure contexts are warranted to validate and extend these results.

## Supplementary Information

Additional file 1: Table S1. Full search strategies and number of records identified from each database. Table S2. Original assessments of analyzed variables and how these variables were harmonized before meta-analyses. Table S3. Detailed definition of ICD-10 codes for cause specific mortality. Table S4. Variables of each cohort studies and data harmonization before conducting the pooled individual participant data analysis. Table S5. Testing the Cox proportional hazards assumption for joint associations of leisure-time physical activity and ambient PM_2.5_ with all-cause mortality. Table S6. Characteristics of prospective studies included in the meta-analysis. Table S7. Quality assessment using the US National Heart, Lung, and Blood Institute (NHLBI) quality assessment tool for observational cohort studies. Table S8. Meta-regression analysis (number of effect estimates = 87). Table S9. Assessment for the certainty of evidence in the meta-analysis. Table S10. Descriptive statistics for leisure-time physical activity engagement at baseline (n = 869,038). Table S11. Independent associations of leisure-time physical activity and ambient PM_2.5_ with all-cause and specific-cause mortality (n = 869,038). Table S12. Sensitivity analysis 1: Joint associations of leisure-time physical activity and ambient PM_2.5_ with all-cause and specific-cause mortality in adults using multiple imputation with further excluding participants who died within the first two years of follow-up among different subgroup populations (n = 865,825). Table S13. Sensitivity analysis 2: Joint associations of leisure-time physical activity (using a new categorization) and ambient PM_2.5_ with all-cause and specific-cause mortality in adults using multiple imputation across different subgroup populations (n = 869,038). Table S14. Sensitivity analysis 3: Joint associations of leisure-time physical activity and ambient PM_2.5_ with all-cause and cause-specific mortality based on complete-case analysis (n = 713,120). Table S15. Multiplicative and additive interactions between leisure-time physical activity and ambient PM_2.5_ on all-cause mortality. Table S16. Numbers of participants and deaths across categories of PM_2.5_ exposure and leisure-time physical activity in the UK and Taiwan cohorts (n = 869,038). Figure S1. The flowchart of the analytical sample selection in the pooled individual participant data analysis. Figure S2. Testing the Cox proportional hazards using scaled Schoenfeld residual. Figure S3. Funnel plot with imputed studies using random effects model. Figure S4. Annual average distribution of ambient PM_2.5_ concentrations in the included cohort data during follow-up periods using box plots. PRISMA 2020 Checklist. STROBE Statement—checklist.

## Data Availability

The study-specific summary data included in the meta-analysis are available from the corresponding author upon reasonable request (Email: ljchen@gm.ntus.edu.tw). The pooled individual participant data that support the analyses of this study are not publicly available; access requires approval through the respective data access procedures of the contributing biobanks. Researchers may apply to obtain these data from the following sources: (1) UK Biobank: https://www.ukbiobank.ac.uk/use-our-data/apply-for-access; (2) Taiwan Biobank: https://www.biobank.org.tw/english.php; (3) MJ Cohort: http://www.mjhrf.org/en/index.php.

## Abbreviations

PM_2.5_: Fine particulate matter with aerodynamic diameter ≤ 2.5 μm
LTPA: Leisure-time physical activity
HR: Hazard ratio
CI: Confidence interval
PRISMA: Preferred Reporting Items for Systematic Reviews and Meta-Analyses
BMI: Body mass index
CVD: Cardiovascular disease
UKB: UK Biobank
TWB: Taiwan Biobank
MET-h/week: Metabolic equivalent hours per week
GBD: Global burden of disease
WHO: World Health Organization
SD: Standard deviation
RR: Relative risk
I.^2^: I-squared statistic (for heterogeneity)
GRADE: Grading of Recommendations, Assessment, Development, and Evaluation
