# Supplementary material for: Does ambient PM2.5 reduce the protective association of leisure-time physical activity with mortality? A systematic review, meta-analysis, and individual-level pooled analysis of cohort studies involving 1.5 million adults
Source: BMC Med. 2025 Nov 28;23:647. doi: 10.1186/s12916-025-04496-y (PMC12661664; doi:10.1186/s12916-025-04496-y)
Supplement: Supplementary file 1 — Additional file 1: Table S1. Full search strategies and number of records identified from each database. Table S2. Original assessments of analyzed variables and how these variables were harmonized before meta-analyses. Table S3. Detailed definition of ICD-10 codes for cause specific mortality. Table S4. Variables of each cohort studies and data harmonization before conducting the pooled individual participant data analysis. Table S5. Testing the Cox proportional hazards assumption for joint associations of leisure-time physical activity and ambient PM2.5 with all-cause mortality. Table S6. Characteristics of prospective studies included in the meta-analysis. Table S7. Quality assessment using the US National Heart, Lung, and Blood Institute (NHLBI) quality assessment tool for observational cohort studies. Table S8. Meta-regression analysis (number of effect estimates = 87). Table S9. Assessment for the certainty of evidence in the meta-analysis. Table S10. Descriptive statistics for leisure-time physical activity engagement at baseline (n = 869,038). Table S11. Independent associations of leisure-time physical activity and ambient PM2.5 with all-cause and specific-cause mortality (n = 869,038). Table S12. Sensitivity analysis 1: Joint associations of leisure-time physical activity and ambient PM2.5 with all-cause and specific-cause mortality in adults using multiple imputation with further excluding participants who died within the first two years of follow-up among different subgroup populations (n = 865,825). Table S13. Sensitivity analysis 2: Joint associations of leisure-time physical activity (using a new categorization) and ambient PM2.5 with all-cause and specific-cause mortality in adults using multiple imputation across different subgroup populations (n = 869,038). Table S14. Sensitivity analysis 3: Joint associations of leisure-time physical activity and ambient PM2.5 with all-cause and cause-specific mortality based on complete-case analysis (n = 713,120 [file 12916_2025_4496_MOESM1_ESM.docx]

**Does ambient PM_2.5_ reduce the protective association of leisure-time physical activity with mortality? A systematic review, meta-analysis, and individual-level pooled analysis of cohort studies involving 1.5 million adults**

**Contents**

**Table S1.** Full search strategies and number of records identified from each database (p.1)

**Table S2**. Original assessments of analyzed variables and how these variables were harmonized before meta-analyses (p.2)

**Table S3.** Detailed definition of ICD-10 codes for cause specific mortality (p.3)

**Table S4.** Variables of each cohort studies and data harmonization before conducting the pooled individual participant data analysis (pp.4–6)

**Table S5****.** Testing the Cox proportional hazards assumption for joint associations of leisure-time physical activity and ambient PM_2.5_ with all-cause mortality (p.7)

**Table S6.** Characteristics of prospective studies included in the meta-analysis (pp.8–14)

**Table S7.** Quality assessment using the US National Heart, Lung, and Blood Institute (NHLBI) quality assessment tool for observational cohort studies (pp. 15-16)

**Table S8.** Meta-regression analysis (number of effect estimates =87) (pp.17–18)

**Table S9.** Assessment for the certainty of evidence in the meta-analysis (p.19)

**Table S10.** Descriptive statistics for leisure-time physical activity engagement at baseline (n=869,038) (p.20)

**Table S11.** Independent associations of leisure-time physical activity and ambient PM_2.5_ with all-cause and specific-cause mortality (n=869,038) (p.21)

**Table S12.** Sensitivity analysis 1: Joint associations of leisure-time physical activity and ambient PM_2.5_ with all-cause and specific-cause mortality in adults using multiple imputation with further excluding participants who died within the first two years of follow-up among different subgroup populations (n=865,825) (pp. 22–23)

**Table S13.** Sensitivity analysis 2: Joint associations of leisure-time physical activity (using a new categorization) and ambient PM_2.5_ with all-cause and specific-cause mortality in adults using multiple imputation across different subgroup populations (n=869,038) (pp. 24-25)

**Table S14**. Sensitivity analysis 3: Joint associations of leisure-time physical activity and ambient PM_2.5_ with all-cause and cause-specific mortality based on complete-case analysis (n=713,120) (pp. 26-27)

**Table S15.** Multiplicative and additive interactions between leisure-time physical activity and ambient PM_2.5_ on all-cause mortality (p.28)

**Table S16.** Numbers of participants and deaths across categories of PM_2.5_ exposure and leisure-time physical activity in the UK and Taiwan cohorts (n = 869,038) (p.29)

**Figure S1.** The flowchart of the analytical sample selection in the pooled individual participant data analysis (p.30)

**Figure S2.** Testing the Cox proportional hazards using scaled Schoenfeld residual (p.31)

**Figure S3.** Funnel plot with imputed studies using random effects model (p.32)

**Figure S4.** Annual average distribution of ambient PM_2.5_ concentrations in the included cohort data during follow-up periods using box plots (p.33)

**PRISMA 2020 Checklist.** (p. 34–36)

**STROBE Statement—checklist.** (p. 37–38)

Table S1. Full Search Strategies for Each Database

| Database | Search string | Records identified | Filters / Limits | Search date |
| --- | --- | --- | --- | --- |
| PubMed | (physical activity OR exercise OR sport OR leisure OR inactivity OR sedentary behavior) AND (mortality OR death OR fatal) AND (air quality OR air pollution OR particulate matter OR fine particle) AND (cohort OR longitudinal OR prospective) AND (Cox OR survival OR hazard OR risk OR relative risk) | 152 | No filters; searched up to 6 Jan 2025 | 6 Jan 2025 |
| Embase | (same as above) | 316 | No filters; searched up to 6 Jan 2025 | 6 Jan 2025 |
| Web of Science | (same as above) | 286 | No filters; searched up to 6 Jan 2025 | 6 Jan 2025 |
| SPORTDiscus | (same as above) | 2 | No filters; searched up to 6 Jan 2025 | 6 Jan 2025 |

**
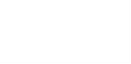
**

**Table S2.** Original assessments of analyzed variables and how these variables were harmonized before meta-analyses

| Cohort | Author (year) | Variables in the original publication | Harmonized variables for meta-analyses |
| --- | --- | --- | --- |
| Published studies | | | |
| US Nurses’ Health Study (NHS)^a^ | Elliott et al. (2020) | LTPA: <3.4, 3.4–10.2, 10.2–22.6, 22.6+ (quartile)(MET-h/wk)  PM_2.5_: Annual mean (quintile)(μg/m^3^)  All-cause mortality | LTPA: No change  PM_2.5_: No change  All-cause mortality (HR): No change |
| Chinese Elderly Health Service (CEHS) | Sun et al. (2020) | LTPA: <1, 1–21, 21+ (MET-h/wk)  PM_2.5_: Annual mean (< 35.3, ≥35.3)(μg/m^3^)  Cardiovascular and respiratory mortality | LTPA: <1, 1–7.5, 7.5–15; 15+ (MET-h/wk)  PM_2.5_: Annual mean (quartile)(μg/m^3^)  All-cause mortality (HR) |
| Taiwan MJ Cohort (MJ) | Guo et al. (2021) | LTPA: 0, 0.1–8.75, 8.75+ (tertile)(MET-h/wk)  PM_2.5_: Annual mean (tertile)(μg/m3)  All-cause mortality | LTPA: <1, 1–7.5, 7.5–15; 15+ (MET-h/wk)  PM_2.5_: Annual mean (quartile)(μg/m^3^)  All-cause mortality (HR): No change |
| US National Health Interview Survey (NHIS) | Coleman et al. (2022) | LTPA: 0, 1–150, 150–300, 300+ min/wk of moderate-intensity activity.  PM_2.5_: Annual mean (quartile)(μg/m^3^)  All-cause mortality | LTPA: <1, 1–7.5, 7.5–15; 15+ (MET-h/wk)  PM_2.5_: Annual mean (quartile)(μg/m^3^)  All-cause mortality (HR): No change |
| Unpublished results | | | |
| Danish Diet, Cancer and Health Cohort (DDCHC)^b^ | Hvidtfeldt & Raaschou-Nielsen (2023) | NA | LTPA: <1, 1–7.5, 7.5–15; 15+ (MET-h/wk)  PM_2.5_: Annual mean (quartile)(μg/m^3^)  All-cause mortality (HR) |
| UK Biobank (UKB) | Xia (2024) | NA | LTPA: <1, 1–7.5, 7.5–15; 15+ (MET-h/wk)  PM_2.5_: Annual mean (quartile)(μg/m^3^)  All-cause mortality (HR) |
| Taiwan Biobank | Ku (2025) |  |  |

HR: hazard ratio; LTPA: leisure-time physical activity; MET-h: metabolic equivalent hours; NA: not applicable; PM_2.5_: fine particulate matter 2.5

a: The effect sizes of the US Nurses’ Health Study (NHS) were not included in the subgroup analyses due to the different coding of exposures.

b: The results was derived from data re-analysis of a published study.

The category ranges of LTPA were defined as lower bound inclusive and upper bound exclusive, except for the highest category (i.e., 15+ MET-h/wk), which was open-ended.”

**Table S3.** Detailed definition of ICD-10 codes for cause specific mortality

| Cause specific mortality | ICD-10 codes |
| --- | --- |
| Cancer | C00-C97, D00-D48 |
| Cardiovascular disease | I01–I02.0, I05–I15, I20–I25, I27, I30–I52, I60–I71 |

ICD: International Classification of Disease

**Table S4.** Variables of each cohort studies and data harmonization before conducting the pooled individual participant data analysis

| **Cohort data**  **Variable** | **UK Biobank** | **Taiwan Biobank** | **Taiwan MJ Cohort** | Harmonized coding |
| --- | --- | --- | --- | --- |
| **1. Enrolment date** | DD/MM/YYYY | DD/MM/YYYY | DD/MM/YYYY | DD/MM/YYYY |
| **2. End of follow-up** | DD/MM/YYYY | DD/MM/YYYY | DD/MM/YYYY | DD/MM/YYYY |
| **Outcome** |  |  |  |  |
| **3. Death case** | 1: yes, 0: no | 1: yes, 0: no | 1: yes, 0: no | 1: yes, 0: no |
| **4. Death dates** | DD/MM/YYYY | DD/MM/YYYY | DD/MM/YYYY | DD/MM/YYYY |
| **Social demographic** |  |  |  |  |
| **5. Country** | 1: UK | 2: Taiwan | 2: Taiwan | from 1 to 3 |
| **6. Age** | years | years | years | years |
| **7. Sex** | 1: male, 2: female | 1: male, 2: female | 1: male, 2: female | 1: male, 2: female |
| **8. Education** | Approximate tertiles- 1: O levels/GCSEs or equivalent /CSEs or equivalent/others. 2: A levels/AS levels or equivalent/NVQ or HND or HNC or equivalent/Other professional qualifications eg: nursing, teaching; 3:  College or University degree. | Approximate tertiles- 1: illiteracy or elementary school; 2: junior high school or senior high school; 3: college/university or above | Approximate tertiles- 1: illiteracy or elementary school; 2: junior high school or senior high school; 3: college/university or above | 1: Low, 2: Medium, 3: High |
| **9.Household income** | Approximate tertiles- 1: < 18,000 GBP; 2: 18,000–52,000 GBP; 3: 52,000+ GBP. | Approximate tertiles and being consistent with Taiwan MJ - 1: < 480,000 NTD; 2: 480,000–960,000 NTD; 3: 960,000+ NTD | Approximate tertiles- 1: < 400,000 NTD; 2: 400,000–800,000 NTD; 3: 800,000+ NTD | 1: Low, 2: Medium, 3: High |
| **Lifestyle behaviors** |  |  |  |  |
| **10. Leisure-time PA** | Self: report: Time per week spent in each type of physical activity was multiplied by each activity’s MET to obtain MET: h/week. | Self: report: Time per week spent in each type of physical activity was multiplied by each activity’s MET to obtain MET: h/week. | Self: report: Time per week spent in each type of physical activity was multiplied by each activity’s MET to obtain MET: h/week. | MET: h/week: 1: <1; 2: 1–7.5; 3: 7.5–15; 4: 15+ |
| **11.Smoking** | 1: former, 2: current, 3: non -smoker | 1: former, 2: current, 3: non-smoker | 1: former, 2: current, 3: non-smoker | 1: former, 2: current, 3: non-smoker |
| **12.Drinking** | 1: current, 2: former, 3: non | 1: former, 2: current, 3: non or <1/wk | 1: former, 2: current, 3: non or <1/wk | 1: current, 2: former, 3: non or occasional |
| **Health status** |  |  |  |  |
| **13.BMI** | BMI (kg/m^2^) | BMI (kg/m^2^) | BMI (kg/m^2^) | BMI (kg/m^2^) |
| **14. Cancer** | 1: yes, 0: no | 1: yes, 0: no | 1: yes, 0: no | 1: yes, 0: no |
| **15. Heart disease** | 1: yes, 0: no | 1: yes, 0: no | 1: yes, 0: no | 1: yes, 0: no |
| **16. Stroke** | 1: yes, 0: no | 1: yes, 0: no | 1: yes, 0: no | 1: yes, 0: no |
| **17. Hypertension** | 1: yes, 0: no | 1: yes, 0: no | 1: yes, 0: no | 1: yes, 0: no |
| **18. Diabetes** | 1: yes, 0: no | 1: yes, 0: no | 1: yes, 0: no | 1: yes, 0: no |
| **19. Liver disease** | 1: yes, 0: no | 1: yes, 0: no | 1: yes, 0: no | 1: yes, 0: no |
| **20. Kidney disease** | 1: yes, 0: no | 1: yes, 0: no | 1: yes, 0: no | 1: yes, 0: no |
| **21. Chronic pulmonary diseases** | 1: yes, 0: no | 1: yes, 0: no | 1: yes, 0: no | 1: yes, 0: no |
| **22. Arthritis** | 1: yes, 0: no | 1: yes, 0: no | 1: yes, 0: no | 1: yes, 0: no |
| **Air pollution (annual mean) over follow-up** |  |  |  |  |
| **23. PM_2.5_** | μg/m3 | μg/m3 | μg/m3 | μg/m3 |

**Table S5.** Testing the Cox proportional hazards assumption for joint associations of leisure-time physical activity and ambient PM_2.5_ with all-cause mortality (n = 869,038)

| Effect | Type 3 test (Joint test) | | |
| --- | --- | --- | --- |
|  | DF | ChiSq (Wald test) | Pr > ChiSq |
| PM_2.5_ & LTPA | 19 | 30839.2317 | < 0.001 |
| Time-dependent variables, *log(time) |  |  |  |
| PM_2.5_ & LTPA | 1 | 2182.7299 | < 0.001 |

ChiSq: Chi-square; DF: degree of freedom; LTPA: leisure-time physical activity

Covariates: Sex, age, educational levels, household income, smoking, alcohol consumption, body mass index, number of chronic diseases, and cohort

**Table S6.** Characteristics of prospective studies included in the meta-analysis

| Author, year,  Country | Study population | Follow-up (year) | LTPA measure | Covariates | PM_2.5_ measure | Mortality | Cox regression/  Interaction effect |
| --- | --- | --- | --- | --- | --- | --- | --- |
| Elliott et al., 2020, US | Cohort: The Nurses’ Health Study  Total n/death= 104990/9827  Age: *M*=63.1, SD=8.9  Proportion of female= 100.0% | 20.0  From baseline (1988) to May 2008. | Self-report: Time per week spent in each LTPA was multiplied by each activity’s MET to obtain MET-h/week.  Quartiles categorization: <3.4 (ref.), 3.4–10.2, 10.2–22.6, and 22.6+. | Age, educational attainment, race, incident cancer, family history of myocardial infarction, smoking, pack-years, alcohol consumption, diet, multivitamin use, census tract median income, Census tract median home value, occupation father, occupation mother, husband’s level of education, registered  nursing degree, marital status, and retirement status. | Residential addresses were updated every 2 years with each questionnaire cycle. Exposure to PM_2.5_ at each residential address using a spatiotemporal prediction model was calculated (24-month  average PM_2.5_). The generalized additive mixed models used monthly average PM_2.5_ monitoring data from  the US EPA’s Air Quality System and other publicly available networks.  Quintiles categorization was based on the annual average of PM_2.5_ during the follow-up period:  Q1: < 10.7 μg/m^3^ (M=9.1, SD=1.3)  Q2: 10.7– 12.4 μg/m^3^ (M=11.6, SD=0.4)  Q3: 12.5– 14.4 μg/m^3^ (M=13.5, SD=0.5)  Q4: 14.4– 16.5 μg/m^3^ (M=15.5, SD=0.5)  Q5: 16.5+ μg/m^3^ (M=18.8, SD=2.0) | Deaths were either reported by the next of kin or through searches of the National Death Index for non–respondents | Time-varying Cox regression with time-varying variables (i.e., PM_2.5_, LTPA, and covariates) on a 1-year (annual) time scale/  P for interaction= 0.60 |
| Sun et al., 2020,  HK (SAR of China) | Cohort: The Chinese Elderly Health Service Cohort  Total n/death= 58643/15874  Age: M= 71.9, SD=5.5  Proportion of female= 65.7% | 10.3  From baseline (1998–2001) to Dec. 2011 | Self-report: Time per week spent in each LTPA was multiplied by each activity’s MET to obtain MET-h/week.  Categorization based on WHO Physical Activity Guidelines:  <1 (ref.), 1–7.5, 7.5–15, 15+. | Age, sex, education, BMI, smoking, alcohol consumption, medication, chronic conditions, personal monthly expenditure, the tertiary planning unit level covariates ((including % of population aged 65+, % with tertiary education and % with income $1923+ USD per month) and smoking rate at district level`), and the smoking rate at the district level. | Satellite-based spatiotemporal model to estimate the average concentration of PM_2.5_ at the residential address of each participant during the follow-up period.  Quartile categorization:  Q1: 26.4– 33.8 μg/m^3^ (M=26.4, SD=1.0)  Q2: 33.8– 35.3 μg/m^3^ (M=34.6, SD=0.4)  Q3: 35.3–37.2 μg/m3 (M=36.2, SD=0.5)  Q4: 37.2– 44.6 μg/m3 (M=38.8, SD=1.3) | Linkage to the death registration in the Department  of Health, Hong Kong | Cox regression/  P for interaction= 0.44 |
| Guo et al., 2021, Taiwan | Cohort: MJ Cohort Study  Total n/death= 384130/12375  Age: M= 39.2, SD=12.7  Proportion of female= 51.3% | 13.4  (median)  From baseline (2001–2016) to 2019 | Self-report: Time per week spent in each LTPA was multiplied by each activity’s MET to obtain MET-h/week.  Categorization based on WHO Physical Activity Guidelines:  <1 (ref.), 1–7.5, 7.5–15, 15+. | Age, sex, educational level, BMI, physical labor at work, smoking, alcohol drinking, vegetable intake, fruit intake, occupational  exposure, chronic diseases (including diabetes, hypertension, dyslipidemia, triglyceride or high-density lipoprotein cholesterol, cardiovascular diseases, and chronic obstructive pulmonary disease), season, and year of enrolment. | Satellite-based spatiotemporal model to estimate the annual concentration of PM_2.5_ at the residential address of each participant. The 2-year average concentration was computed based on the year of medical examination and the previous year.  Quartile categorization was based on the annual average of PM_2.5_ during the follow-up period:  Q1: < 21.57 μg/m^3^ (M=19.3, SD=2.0)  Q2: 21.57– 23.93 μg/m^3^ (M= 22.8, SD=0.7)  Q3: 23.93 –27.80 μg/m^3^(M=25.5, SD=1.0)  Q4: ≥ 27.80 μg/m^3^ (M= 37.6, SD=5.3) | Linkage to Taiwan National Death Registry | Time-varying Cox regression with time-varying variables (i.e., PM_2.5_, LTPA, and covariates) on a 1-year (annual) time scale/  P-value for interaction=0.03 |
| Coleman et al., 2022, US | Cohort: The National Health Interview Survey (NHIS) data  Total n/death= 403748/39528  Age: *M*=45.9, SD=17.0  Proportion of female= 54.7% | 7.0  Baseline (1997–2014 NHIS data) till 2015 | Self-report:  Time per week spent in moderate and vigorous LTPA was collected. Four levels of moderate LTPA were classified: (0 min/wk), insufﬁciently active (0.1–150 min/wk), sufﬁciently active (150–300 min/wk), and highly active (300+ min/wk).  Categorization based on WHO Physical Activity Guidelines:  <1 (ref.), 1–7.5, 7.5–15, 15+. | Age, sex, education, household income, ethnicity, marital status, smoking, physical activity, BMI, urbanization, census region, survey year, ecoregion, NDVI | Census tract-level estimates for PM_2.5_ were obtained using integrated ground-based monitored data with satellite-derived estimates of land use and air pollution, along with other land use and geographic variables. Averaged 1999–2015 PM_2.5_ estimates were used for this study.  Quartile categorization:  Q1: < 9.1 μg/m^3^ (M=7.9, SD= NR)  Q2: 9.1– 10.6 μg/m^3^ (M=9.7, SD=NR)  Q3: 10.6 – 12.2 μg/m^3^ (M=11.4, SD=NR)  Q 4: 12.2+ μg/m^3^ (M=13.4, SD=NR) | Linkage to the US National Death Index | Cox regression/  P for interaction > 0.05 |
| Unpublished results, Hvidtfeldt and Raaschou-Nielsen, 2023, Denmark | Cohort: The Danish Diet, Cancer and Health cohort  Total n/death= 49,560/10,190  Age: *Median*=56.3, *Mean*=56.7, SD=4.4  Proportion of female= 53.1% | 18.1  From baseline (1993–1997) to 2015 | Self-report: Time per week spent in each LTPA (walking, cycling, gardening, and sports) was multiplied by each activity’s MET to obtain MET-h/week  Categorization based on WHO Physical Activity Guidelines:  <1 (ref.), 1–7.5, 7.5-15, 15+. | Age, sex, occupational status, educational attainment, marital status, BMI, alcohol consumption, smoking, fruit consumption, vegetable  consumption, and neighborhood-level socioeconomic status (SES). | PM_2.5_ exposure concentrations as an annual average during the follow-up period based on yearly concentrations at all addresses  Quartile categorization:  Q1: 11.0– 14.1 μg/m^3^ (M=13.8, SD=0.2)  Q2: 14.1– 14.5 μg/m^3^ (M=14.3, SD=0.1)  Q3: 14.5– 15.3 μg/m^3^ (M=14.8, SD=0.2)  Q4: 15.3– 36.4 μg/m^3^ (M=16.7, SD=1.6) | Linkage to the Civil Registration System, which includes date of emigration and death, and the Danish Register of Causes of Death | Cox regression/  P for interaction = 0.84 |
| Unpublished results, Xia, 2024, UK | Cohort: The UK Biobank  Total n/death= 453,732/33,249  Age: *M*=56.6, SD=8.1  Proportion of female= 54.3% | 12.3  SD=1.76  From baseline (2006–2010) to Dec. 2023 | Self-report:  Frequency and duration spent in LTPA, including walking for pleasure, other exercises and strenuous sports, were reported to compute. MET-h/week  Categorization based on WHO Physical Activity Guidelines:  <1 (ref.), 1–7.5, 7.5–15, 15+ | Age, sex, education, ethnicity, household income, employment status, smoking, alcohol intake, BMI and chronic conditions ((hypertension, diabetes, cardiovascular disease, and cancer). | The annual average concentrations of PM_2.5_ in 2010 were computed via a land use regression model and linked to the geocoded residential addresses of the participants.  Quartile categorization:  Q1: 8.2– 9.3 μg/m^3^ (M=8.7, SD=0.4)  Q2: 9.3– 9.9 μg/m^3^ (M=9.6, SD=0.2)  Q3: 9.9– 10.6 μg/m^3^ (M=10.2, SD=0.2)  Q4: 10.6– 21.3 μg/m^3^ (M=11.4, SD=0.8) | Linkage to the UK National Health Services register | Cox regression/  P for interaction= 0.40 |
| Unpublished results, Ku, 2025, Taiwan | Cohort: Taiwan Biobank  Total n/death= 135,261/1,218  Age: Median=51.0, Mean=49.7, SD=11.0  Proportion of female= 63.8% | 5.2  SD=2.5  From baseline (2008–2020) to Dec. 2021 | Self-report: Time per week spent in each LTPA was multiplied by each activity’s MET to obtain MET-h/week.  Categorization based on WHO Physical Activity Guidelines:  <1 (ref.), 1–7.5, 7.5–15, 15+. | Age, sex, education, alcohol intake, and chronic conditions (heart disease, stroke, hypertension, diabetes, and cancer). | The PM_2.5_ monitoring data was acquired from the datasets of Environmental Protection Administration, Executive Yuan (<https://data.epa.gov.tw>). In the Taiwan Biobank comprehensive residential addresses of respondents were not made publicly available. Instead, annual average estimates were computed utilizing the Ordinary Kriging Model based on the residential areas (townships/cities and districts) of participants throughout Taiwan. Yearly mean concentrations specific to the geocoded participants' addresses were estimated for the period between 2008 and 2020 through 76 nationwide atmospheric monitoring sites in Taiwan.  Quartile categorization:  Q1: 6.6– 15.2 μg/m^3^ (M=12.6, SD=1.9)  Q2: 15.2–18.8 μg/m^3^ (M=17.0, SD=1.0)  Q3: 18.8–22.9 μg/m^3^ (M=20.7, SD=1.2)  Q4: 22.9–40.8 μg/m^3^ (M=25.7, SD=2.1) | Linkage to Taiwan National Death Registry | Cox regression/  P for interaction < 0.001 |

BMI: body mass index; HK (SAR): Hong Kong (Special Administrative Region of China); HR: hazard ratio; LTPA: leisure-time physical activity; M: Mean; MET-h: metabolic equivalent hours; NDVI: normalized difference vegetation index; NR: nor reported; PM_2.5_: fine particulate matter 2.5; Q: quartile (quintile in Elliont et al., 2020); SD: standard deviation; WHO: World Health Organization

The maximally adjusted HRs from multivariable proportional hazards models were selected to alleviate the potential confounding bias in each study.

The category ranges of PM_2.5_ and LTPA were defined as lower bound inclusive and upper bound exclusive, except for the highest category (i.e., 15+ MET-h/wk), which was open-ended.”

**Table S7.** Quality assessment using the US National Heart, Lung, and Blood Institute (NHLBI) quality assessment tool for observational cohort studies

| **Author (Year)** | | **Raters** | **Quality assessment criteria** | | | | | | | | | | | | | | **Total Score (percentage)** | **Quality Rating** |  |
| --- | --- | --- | --- | --- | --- | --- | --- | --- | --- | --- | --- | --- | --- | --- | --- | --- | --- | --- | --- |
|  |  |  | **1** | **2** | **3** | **4** | **5** | **6** | **7** | **8** | **9** | **10** | **11** | **12** | **13** | **14** |  |  |  |
| Elliot et al. (2020)  US | | YX  BY | Y  Y | Y  Y | Y  Y | Y  Y | N  N | Y  Y | Y  Y | Y  Y | Y  Y | Y  Y | Y  N | CD  CD | Y  Y | Y  Y | 11/14 (78.6%) | Good |  |
|  | | LC |  |  |  |  |  |  |  |  |  |  | N |  |  |  |  |  |  |
| Sun et al. (2020)  HK (SAR of China) | | YX  BY | Y  Y | N  N | NR  Y | Y  Y | N  N | Y  Y | Y  Y | Y  Y | Y  Y | N  N | Y  Y | Y  Y | Y  Y | Y  Y | 11/14 (78.6%) | Good |  |
|  | | LC |  |  | Y |  |  |  |  |  |  |  |  |  |  |  |  |  |  |
| Guo et al. (2021)  Taiwan | | YX  BY | Y  Y | N  N | NR  Y | N  N | N  N | Y  Y | Y  Y | Y  Y | Y  Y | Y  NR | Y  Y | NR  Y | Y  Y | Y  Y | 10/14 (71.4%) | Fair |  |
|  | | LC |  |  | Y |  |  |  |  |  |  | NR |  | Y |  |  |  |  |  |
| Coleman et al. (2022)  US | | YX  BY | Y  Y | Y  Y | Y  Y | Y  Y | N  N | Y  Y | Y  N | Y  Y | Y  Y | N  Y | Y  Y | Y  Y | Y  Y | N  N | 10/14 (71.4%) | Fair |  |
|  | | LC |  |  |  |  |  |  | CD |  |  | N |  |  |  |  |  |  |  |
| Hvidtfeldt & Raaschou-Nielsen (2023) DDCHC, Denmark | | YX  BY | Y  Y | Y  Y | N  N | Y  Y | N  N | Y  Y | Y  Y | Y  Y | Y  Y | N  N | Y  Y | Y  Y | Y  Y | Y  Y | 11/14 (78.6%) | Good |  |
| Xia (2023) UK Biobank | | YX  BY | Y  Y | N  N | Y  Y | Y  Y | N  N | Y  Y | Y  Y | Y  Y | Y  Y | N  N | Y  Y | Y  Y | Y  Y | Y  Y | 11/14 (78.6%) | Good |  |
| Ku (2025) Taiwan Biobank | | YX  BY | Y  Y | N  N | Y  Y | N  N | N  N | Y  Y | Y  Y | Y  Y | Y  Y | N  N | Y  Y | Y  Y | Y  Y | Y  Y | 10/14 (71.4) | Fair |  |
|  | The National Institutes of Health (NIH) Study Quality Assessment Tool **(**https://www.nhlbi.nih.gov/health-topics/study-quality-assessment-tools**)**  DDCHC: Danish Diet, Cancer and Health Cohort; CFPS: China Family Panel Studies  The 14 Criteria: **1**. Was the research question or objective in this paper clearly stated? **2**. Was the study population clearly specified and defined? **3**. Was the participation rate of eligible persons at least 50%? **4**. Were all the subjects selected or recruited from the same or similar populations (including the same time period)? Were inclusion and exclusion criteria for being in the study prespecified and applied uniformly to all participants? **5**. Was a sample size justification, power description, or variance and effect estimates provided? **6**. For the analyses in this paper, were the exposure(s) of interest measured prior to the outcome(s) being measured? **7**. Was the timeframe sufficient so that one could reasonably expect to see an association between exposure and outcome if it existed? **8**. For exposures that can vary in amount or level, did the study examine different levels of the exposure as related to the outcome (e.g., categories of exposure, or exposure measured as a continuous variable)? **9**. Were the exposure measures (independent variables) clearly defined, valid, reliable, and implemented consistently across all study participants? **10**. Was the exposure(s) assessed more than once over time? **11**. Were the outcome measures (dependent variables) clearly defined, valid, reliable, and implemented consistently across all study participants? **12**. Were the outcome assessors blinded to the exposure status of participants? **13**. Was loss to follow-up after baseline 20% or less? **14**. Were key potential confounding variables measured and adjusted statistically for their impact on the relationship between exposure(s) and outcome(s)?  CFPS: China Family Panel Studies; Danish Diet, DDCHC: Cancer and Health Cohort; NHIS: National Health Interview Survey  **N:** no; **Y:** yes.**CD:** cannot be determined; **NA:** not applicable; **NR:** not reported; Percentage: **Total Score:** This represents the total count of criteria rated as 'Yes.'  **Percentage:** This is the proportion of criteria, out of the 14 evaluated, that have been rated as 'Yes.  **Quality Rating:** **Poor** (total score <7, < 50%); **Fair** (total score 7–11, 50–75%; **Good** (total score 11+, ≥75%)  Note: Two authors (YX and BY) independently conducted the work, and any discrepancies were resolved through consensus. In cases of unresolved disagreements, a third author (LC) was consulted for a final decision. Research quality was rated based on the scores: Poor (< 7), Fair (scores 7–11), or Good (scores 11+), with the highest possible score being 14.^1^ | | | | | | | | | | | | | | | | | | |

**Table S8.** Meta-regression analysis (number of effect estimates =87)

| Covariates | Coefficients (95%CI) | *p*-values | R^2^ analog. |
| --- | --- | --- | --- |
| Model 1 |  |  | 0.60 |
| Intercept | -0.18 (-0.27, -0.09) | < 0.001 |  |
| LTPA (MET-h/week)^a^ | -0.15 (-0.19, -0.11) | < 0.001 |  |
| Levels of PM_2.5_ (μg/m^3^) |  | < 0.001 |  |
| < 10 (ref.) | 0.00 |  |  |
| 10–15 | -0.005 (-0.01, 0.09) | 0.92 |  |
| 15–25 | -0.02 (-0.12, 0.09) | 0.72 |  |
| 25–35 | 0.23 (0.12, 0.35) | < 0.001 |  |
| 35–50 | 0.21 (0.09, 0.33) | < 0.001 |  |
| Model 2 |  |  | 0.94 |
| Intercept | -0.26 (-0.30, -0.22) | < 0.001 |  |
| LTPA (MET-h/week)^a^ | -0.11 (-0.13, -0.09) | < 0.001 |  |
| Levels of PM_2.5_ (μg/m^3^) |  | < 0.001 |  |
| < 10 (ref.) | 0.00 |  |  |
| 10–15 | 0.04 (-0.005, 0.09) | 0.08 |  |
| 15–25 | 0.06 (0.01, 0.12) | 0.03 |  |
| 25–35 | 0.20 (0.14, 0.26) | < 0.001 |  |
| 35–50 | 0.17 (0.11, 0.22) | < 0.001 |  |
| Mean age of samples (years) | 0.01 (0.01, 0.01) | < 0.001 |  |
| Proportion of females (%) | -0.01 (-0.01, -0.01) | < 0.001 |  |
| Model 3 |  |  | 0.94 |
| Intercept | -0.26 (-0.30, -0.22) | < 0.001 |  |
| LTPA (MET-h/week)^a^ | -0.11 (-0.13, -0.09) | < 0.001 |  |
| Levels of PM_2.5_ (μg/m^3^) |  | < 0.001 |  |
| < 10 (ref.) | 0.00 |  |  |
| 10–15 | 0.04 (-0.01, 0.08) | 0.08 |  |
| 15–25 | 0.07 (0.01, 0.13) | 0.03 |  |
| 25–35 | 0.20 (0.14, 0.26) | < 0.001 |  |
| 35–50 | 0.17 (0.11, 0.22) | < 0.001 |  |
| Mean age of samples (years) | 0.01 (0.01, 0.01) | < 0.001 |  |
| Proportion of females (%) | -0.01 (-0.01, -0.01) | < 0.001 |  |
| Mean length of follow-up (years) | -0.001 (-0.01, 0.01) | 0.71 |  |
| Model 4 |  |  | 0.95 |
| Intercept | -0.25 (-0.30, -0.21) | < 0.001 |  |
| LTPA (MET-h/week)^a^ | -0.11 (-0.13, -0.09) | < 0.001 |  |
| Levels of PM_2.5_ (μg/m^3^) |  | < 0.001 |  |
| < 10 (ref.) | 0.00 |  |  |
| 10–15 | 0.04 (-0.01, 0.08) | 0.12 |  |
| 15–25 | 0.06 (0.001, 0.11) | 0.05 |  |
| 25–35 | 0.17 (0.10, 0.24) | < 0.001 |  |
| 35–50 | 0.14 (0.07, 0.21) | < 0.001 |  |
| Mean age of studies | 0.01 (0.01, 0.01) | < 0.001 |  |
| Proportion of females | -0.01 (-0.01, -0.01) | < 0.001 |  |
| Publication (unpublished [ref.]) | 0.04 (-0.02, 0.09) | 0.18 |  |

LTPA: leisure-time physical activity; MET-h: metabolic equivalent hours; PM_2.5_: fine particulate matter 2.5

a: LTPA was coded as a continuous variable by an increment of 7.5 MET-h per week.

Model 3: based on Model 2, further including ‘mean length of follow-up’.

Model 4: based on Model 2, further including ‘publication status’.

Test for linear trend: PM_2.5_ was further coded as a continuous variable ranging from 1 to 5 (i.e., < 10 to 35-50 μg/m^3^): *p*-values for all models < 0.001.

The category ranges of PM_2.5_ were defined as lower bound inclusive and upper bound exclusive.

Note:

1. LTPA categories was transformed into scores of MET-h per week based on the midpoint of the range in each category. For example, “1-<7.5 MET-h/week” was coded as 4.25 MET-h/week.
2. All variables, except LTPA and PM2.5, were transformed via mean centering before entering the meta-regression models. Based on the criteria of variance inﬂation factor >5 (or tolerance <0.2) and condition indices >30. ^2, 3^

**Table S9.** Assessment for the certainty of evidence in the meta-analysis

| No of studies  Total n/death | Downgrade | | | |  |  | Upgrade | | |  | Certainty of evidence |
| --- | --- | --- | --- | --- | --- | --- | --- | --- | --- | --- | --- |
| 7 cohort studies;  Total n/death= 1,515,094/  115,196 | Risk of bias | Inconsistency | Indirectness | Imprecision | Publication bias |  | Large effect | Dose-response | Residual confounding |  |  |
|  | (0)  No downgrading was applied, given that most studies were rated as good quality. | (-1)  Although we harmonized the data, the heterogeneity across studies remained high because the effect sizes were derived from different levels of LTPA within the quartiles of each study. This was supported by the high R^2^ analogue in the meta-regression. | (0)  No downgrading was applied. The research question in the studies reflected the primary question of the meta-analysis due to data re-analysis and variable harmonization. | (0)  No downgrading was applied because the sample sizes of the studies were relatively large, and the number of mortality cases used for calculating the pooled effect size was higher than 100,000. | (0)  No downgrading was applied as no evidence of publication bias was found in funnel plots and Egger’s tests. |  | (0)  The effect sizes were not large. | (+1)  There is evidence of gradient in the meta-analysis | (0)  Unmeasured confounding could influence the effect estimate. |  | Moderate (⊕⊕⊕) |

This meta-analysis involved large-scale prospective studies of fair to good quality. Therefore, we started the rating process with moderate certainty.

Grades for the certainty of evidence: high (⊕⊕⊕⊕); moderate (⊕⊕⊕); low (⊕⊕); very low (⊕).

Based on the quality assessment of included studies in Table A7 (which ranged from fair to good), the initial quality rating of the body of evidence was considered moderate (⊕⊕⊕).

Note: The approach to rating the quality of evidence starts with the study design (trials or observational studies) and then addresses ﬁve reasons (1. Risk of bias; 2. Inconsistency; 3. Indirectness; 4. Imprecision; 5. Publication bias.) to possibly downgrade the quality of evidence and three (1. Large magnitude of effect size; 2. All plausible residual confounding; 3. Dose-response gradient.) to possibly upgrade the quality.^4, 5^

**Table S10.** Descriptive statistics for leisure-time physical activity engagement at baseline (n=869,038)

| Variables | n | Leisure-time physical activity (MET-h/wk)(%) | | | | |
| --- | --- | --- | --- | --- | --- | --- |
|  |  | <1 | 1–7.5 | 7.5–15 | 15+ | *P-value* for χ^2^ |
| **Socio-demographic** |  |  |  |  |  |  |
| Sex |  |  |  |  |  | < 0.001 |
| Male | 389,337 | 25.3 | 35.3 | 15.2 | 24.3 |  |
| Female | 479,701 | 27.4 | 36.9 | 15.8 | 19.9 |  |
| Age |  |  |  |  |  | < 0.001 |
| 65+ | 118,129 | 24.5 | 27.5 | 18.4 | 29.6 |  |
| 45-65 | 481,097 | 27.0 | 29.6 | 18.0 | 25.3 |  |
| 18-45 | 269,812 | 26.4 | 51.4 | 9.9 | 12.3 |  |
| Educational level^a^ |  |  |  |  |  | < 0.001 |
| Low | 261,385 | 27.3 | 31.6 | 17.4 | 23.8 |  |
| Medium | 352,073 | 21.6 | 37.5 | 17.2 | 23.7 |  |
| High | 255,580 | 26.9 | 41.6 | 12.5 | 19.0 |  |
| Household annual income^a^ |  |  |  |  |  | < 0.001 |
| Low | 225,133 | 33.2 | 33.9 | 13.2 | 19.7 |  |
| Medium | 373,973 | 23.9 | 35.5 | 16.9 | 23.7 |  |
| High | 269,932 | 20.9 | 41.7 | 15.9 | 21.6 |  |
| Cohort |  |  |  |  |  | < 0.001 |
| UK Biobank | 501,088 | 23.0 | 31.0 | 19.5 | 26.4 |  |
| Taiwan Biobank | 135,261 | 60.0 | 3.6 | 10.1 | 26.3 |  |
| MJ Cohort | 232,689 | 14.3 | 66.0 | 10.3 | 9.5 |  |
| Health-related behaviors |  |  |  |  |  |  |
| Smoking |  |  |  |  |  | < 0.001 |
| Former | 201,851 | 23.3 | 30.6 | 18.7 | 27.4 |  |
| Current | 111,571 | 30.7 | 42.4 | 11.6 | 15.4 |  |
| Never | 555,616 | 24.8 | 37.8 | 15.6 | 21.7 |  |
| Alcohol consumption |  |  |  |  |  | < 0.001 |
| Former | 27,678 | 32.8 | 31.7 | 13.9 | 21.6 |  |
| Current | 501,237 | 21.7 | 32.9 | 19.2 | 26.2 |  |
| None/occasional | 340,123 | 30.0 | 42.7 | 10.8 | 16.5 |  |
| Health Status |  |  |  |  |  |  |
| Number of chronic diseases |  |  |  |  |  | < 0.001 |
| 2+ | 84,554 | 33.7 | 28.2 | 15.9 | 22.1 |  |
| 1 | 218,864 | 26.7 | 31.6 | 17.4 | 24.3 |  |
| 0 | 565,620 | 25.3 | 39.1 | 14.8 | 20.8 |  |
| Body mass index^b^ |  |  |  |  |  | < 0.001 |
| Obese | 144,662 | 34.5 | 32.8 | 15.4 | 17.3 |  |
| Overweight | 306,328 | 25.4 | 33.2 | 17.2 | 24.2 |  |
| Normal | 389,884 | 24.3 | 38.2 | 14.9 | 22.6 |  |
| Underweight | 28,164 | 23.9 | 59.0 | 8.1 | 8.9 |  |
| Annual mean of PM_2.5_ (μg/m^3^) |  |  |  |  |  | < 0.001 |
| 35–50 | 14,583 | 14.0 | 63.0 | 11.5 | 11.5 |  |
| 25–35 | 71,416 | 23.1 | 50.9 | 10.9 | 15.2 |  |
| 15–25 | 250,593 | 30.8 | 44.5 | 10.1 | 14.7 |  |
| 10–15 | 263,641 | 29.9 | 28.0 | 17.6 | 24.5 |  |
| <10 | 268,805 | 21.1 | 30.7 | 20.1 | 28.2 |  |

MET-h, metabolic equivalent hours; PM_2.5_: fine particulate matter 2.5

a: Details on the original categories and data harmonization can be found in Table A6.

b: Cut-offs of body mass index (underweight, normal, overweight, and obese) were based on country-specific recommendations: UK (< 18.5, 18.5– 23.0, 23.0– 25.0, 25.0+), and Taiwan (< 18.5, 18.5–24.0, 24.0–27.0, 27.0+).

The category ranges of age, LTPA, and body mass index were defined as lower bound inclusive and upper bound exclusive, except for the highest categories, which was open-ended.

**Table A11.** Independent associations of leisure-time physical activity and ambient PM_2.5_ with all-cause and specific-cause mortality (n=869,038)

| Variables | n | All-cause Mortality^a^ | | | Cancer Mortality^a, b^ | | | Cardiovascular Mortality^a, b^ | | | |
| --- | --- | --- | --- | --- | --- | --- | --- | --- | --- | --- | --- |
|  |  | Deaths | aHRs | 95% CI | Deaths | aHRs | 95% CI | | Deaths | aHRs | 95% CI |
| LTPA (MET-h/week) ^c^ |  |  |  | p < 0.001 |  |  | p < 0.001 | |  |  | p < 0.001 |
| < 1 (Reference) | 229,890 | 14,700 | 1.00 |  | 6,364 | 1.00 |  | | 3,081 | 1.00 |  |
| 1–7.5 | 313,974 | 13,849 | **0.77** | **0.75–0.79** | 7,095 | **0.87** | **0.84–0.90** | | 2,444 | **0.72** | **0.68–0.76** |
| 7.5–<15 | 135,168 | 7,123 | **0.73** | **0.70–0.75** | 3,790 | **0.84** | **0.81–0.87** | | 1,300 | **0.71** | **0.67–0.76** |
| 15+ | 190,006 | 9,408 | **0.69** | **0.67–0.71** | 4,881 | **0.78** | **0.75–0.81** | | 1,785 | **0.70** | **0.66–0.74** |
| PM_2.5_ (μg/m^3^) ^c^ |  |  |  | p < 0.001 |  |  | p < 0.001 | |  |  | p < 0.001 |
| 35–50 (Reference) | 14,583 | 1,033 | 1.00 |  | 448 | 1.00 |  | | 166 | 1.00 |  |
| 25–35 | 71,416 | 3,380 | **0.92** | **0.86–0.99** | 1,489 | 0.93 | 0.83–1.03 | | 585 | 0.96 | 0.80–1.14 |
| 15–25 | 250,593 | 3,361 | **0.44** | **0.38–0.52** | 1,444 | **0.41** | **0.32–0.51** | | 704 | **0.58** | **0.41–0.82** |
| 10–15 | 263,641 | 17,312 | **0.40** | **0.37–0.42** | 8,385 | **0.38** | **0.34–0.42** | | 3,452 | **0.50** | **0.42–0.60** |
| < 10 | 268,805 | 16,714 | **0.41** | **0.35–0.48** | 8,747 | **0.39** | **0.31–0.50** | | 3,087 | **0.52** | **0.37–0.74** |

aHR: adjusted hazard ratio; CI: confidence interval; LTPA: leisure-time physical activity; MET-h: metabolic equivalent hours; PM_2.5_: fine particulate matter 2.5

Bold values indicate statistical significance.

a: Adjusted for sex, age, educational levels, household income, smoking, alcohol consumption, body mass index, number of chronic diseases, and cohort.

b: Using cause-specific hazard models for competing risks of other mortality causes.

c: Test for linear trend: both p-values for LTPA and PM_2.5_ < 0.001

The category ranges of LTPA and PM_2.5_ were defined as lower bound inclusive and upper bound exclusive, except for the highest category (15+ MET-h/wk), which was open-ended.

**Table S12.** Sensitivity analysis 1: Joint associations of leisure-time physical activity and ambient PM_2.5_ with all-cause and specific-cause mortality in adults using multiple imputation with further excluding participants who died within the first two years of follow-up across different subgroup populations (n=865,825)

| Population | PM_2.5_ (μg/m^3^) | LTPA  (MET-h/week) | All-cause Mortality^a^ | | Cancer Mortality^a, b^ | | Cardiovascular Mortality^a, b^ | |
| --- | --- | --- | --- | --- | --- | --- | --- | --- |
|  |  |  | Death | aHR (95% CI) | Death | aHR (95% CI) | Death | aHR (95% CI) |
| Total |  |  |  |  |  |  |  |  |
| n=865,825 | 35–50 | <1 | 42,591 | Ref | 20,794 | Ref | 8,095 | Ref |
|  | 35–50 | 7.5+ |  | **0.80 (0.66, 0.96)** |  | 1.13 (0.85, 1.51) |  | **0.54 (0.35, 0.84)** |
|  | 25–35 | 7.5+ |  | **0.67 (0.58, 0.79)** |  | 0.85 (0.66, 1.10) |  | **0.52 (0.37, 0.74)** |
|  | 15–25 | 7.5+ |  | **0.34 (0.29, 0.39)** |  | **0.43 (0.33, 0.55)** |  | **0.32 (0.22, 0.45)** |
|  | 10–15 | 7.5+ |  | **0.39 (0.31, 0.48)** |  | **0.49 (0.35, 0.68)** |  | **0.36 (0.22, 0.58)** |
|  | <10 | 7.5+ |  | **0.35 (0.28, 0.44)** |  | **0.46 (0.33, 0.65)** |  | **0.32 (0.20, 0.52)** |
| Subgroup |  |  |  |  |  |  |  |  |
| Sex |  |  |  |  |  |  |  |  |
| Female | 35–50 | <1 | 17,537 | Ref | 9,608 | Ref | 2,478 | Ref |
| (n=478,534) | 35–50 | 7.5+ |  | 0.77 (0.58, 1.01) |  | 1.29 (0.85, 1.95) |  | 0.64 (0.32, 1.31) |
|  | 25–35 | 7.5+ |  | **0.60 (0.48, 0.76)** |  | 0.73 (0.50, 1.06) |  | **0.49 (0.27, 0.88)** |
|  | 15–25 | 7.5+ |  | **0.31 (0.25, 0.40)** |  | **0.41 (0.28, 0.59)** |  | **0.33 (0.19, 0.58)** |
|  | 10–15 | 7.5+ |  | **0.34 (0.25, 0.47)** |  | **0.40 (0.24, 0.65)** |  | 0.48 (0.22, 1.08) |
|  | <10 | 7.5+ |  | **0.32 (0.23, 0.44)** |  | **0.38 (0.23, 0.62)** |  | **0.44 (0.20, 0.99)** |
| Male | 35–50 | <1 | 25,054 | Ref | 11,186 | Ref | 5,617 | Ref |
| (n=387,291) | 35–50 | 7.5+ |  | 0.84 (0.65, 1.07) |  | 1.03 (0.68, 1.56) |  | **0.51 (0.29, 0.90)** |
|  | 25–35 | 7.5+ |  | **0.75 (0.60, 0.92)** |  | 0.95 (0.66, 1.36) |  | **0.57 (0.36, 0.89)** |
|  | 15–25 | 7.5+ |  | **0.36 (0.29, 0.45)** |  | **0.44 (0.31, 0.64)** |  | **0.32 (0.21, 0.49)** |
|  | 10–15 | 7.5+ |  | **0.42 (0.31, 0.56)** |  | **0.57 (0.36, 0.90)** |  | **0.32 (0.18, 0.59)** |
|  | <10 | 7.5+ |  | **0.38 (0.28, 0.51)** |  | **0.53 (0.34, 0.85)** |  | **0.28 (0.15, 0.52)** |
| Age |  |  |  |  |  |  |  |  |
| 65+ | 35–50 | <1 | 17,442 | Ref | 7,630 | Ref | 3,583 | Ref |
| (n=116,985) | 35–50 | 7.5+ |  | **0.73 (0.56, 0.97)** |  | 1.22 (0.76, 1.97) |  | 0.58 (0.31, 1.09) |
|  | 25–35 | 7.5+ |  | **0.63 (0.50, 0.81)** |  | 0.76 (0.49, 1.18) |  | **0.53 (0.32, 0.90)** |
|  | 15–25 | 7.5+ |  | **0.30 (0.24, 0.38)** |  | **0.37 (0.24, 0.56)** |  | **0.32 (0.19, 0.54)** |
|  | 10–15 | 7.5+ |  | **0.36 (0.25, 0.53)** |  | **0.45 (0.25, 0.81)** |  | **0.32 (0.14, 0.73)** |
|  | <10 | 7.5+ |  | **0.34 (0.23, 0.49)** |  | **0.43 (0.24, 0.78)** |  | **0.30 (0.13, 0.69)** |
| 18-64 | 35–50 | <1 | 25,149 | Ref | 13,164 | Ref | 4,512 | Ref |
| (n=748,840) | 35–50 | 7.5+ |  | 0.89 (0.70, 1.14) |  | 1.18 (0.82, 1.70) |  | **0.49 (0.26, 0.93)** |
|  | 25–35 | 7.5+ |  | 0.81 (0.66, 1.00) |  | 1.06 (0.77, 1.45) |  | **0.59 (0.36, 0.95)** |
|  | 15–25 | 7.5+ |  | **0.39 (0.32, 0.48)** |  | **0.50 (0.37, 0.69)** |  | **0.33 (0.21, 0.52)** |
|  | 10–15 | 7.5+ |  | **0.41 (0.32, 0.54)** |  | **0.53 (0.35, 0.79)** |  | **0.39 (0.21, 0.70)** |
|  | <10 | 7.5+ |  | **0.40 (0.31, 0.52)** |  | **0.53 (0.36, 0.80)** |  | **0.35 (0.19, 0.64)** |
| CVDs |  |  |  |  |  |  |  |  |
| Yes | 35–50 | <1 | 19,538 | Ref | 8,094 | Ref | 4,895 | Ref |
| (n=192,281) | 35–50 | 7.5+ |  | 0.85 (0.59, 1.21) |  | 1.27 (0.70, 2.32) |  | 0.64 (0.32, 1.28) |
|  | 25–35 | 7.5+ |  | **0.66 (0.48, 0.90)** |  | 0.72 (0.41, 1.26) |  | **0.42 (0.23, 0.76)** |
|  | 15–25 | 7.5+ |  | **0.34 (0.25, 0.46)** |  | **0.37 (0.21, 0.64)** |  | **0.29 (0.16, 0.52)** |
|  | 10–15 | 7.5+ |  | **0.39 (0.27, 0.58)** |  | **0.38 (0.20, 0.73)** |  | **0.37 (0.18, 0.77)** |
|  | <10 | 7.5+ |  | **0.36 (0.24, 0.53)** |  | **0.36 (0.18, 0.69)** |  | **0.33 (0.16, 0.68)** |
| No | 35–50 | <1 | 23,053 | Ref | 12,700 | Ref | 3,200 | Ref |
| (n=673,544) | 35–50 | 7.5+ |  | **0.76 (0.61, 0.95)** |  | 1.06 (0.76, 1.49) |  | **0.43 (0.23, 0.79)** |
|  | 25–35 | 7.5+ |  | **0.68 (0.57, 0.82)** |  | 0.90 (0.67, 1.21) |  | 0.64 (0.41, 1.00) |
|  | 15–25 | 7.5+ |  | **0.34 (0.28, 0.40)** |  | **0.45 (0.34, 0.61)** |  | **0.34 (0.22, 0.53)** |
|  | 10–15 | 7.5+ |  | **0.38 (0.29, 0.50)** |  | **0.55 (0.37, 0.82)** |  | **0.33 (0.17, 0.63)** |
|  | <10 | 7.5+ |  | **0.35 (0.27, 0.45)** |  | **0.52 (0.35, 0.78)** |  | **0.29 (0.15, 0.57)** |

aHR: adjusted hazard ratio; CI: confidence interval; CVDs: cardiovascular diseases; LTPA, leisure-time physical activity; MET-h, metabolic equivalent hours; PM_2.5_: fine particulate matter 2.5

Bold values indicate statistical significance.

a: Adjusted for sex, age, educational levels, household income, smoking, alcohol consumption, body mass index, number of chronic diseases, and cohort.

b: Using cause-specific hazard models for competing risks of other mortality causes.

The category ranges of PM_2.5_ and LTPA were defined as lower bound inclusive and upper bound exclusive, except for the highest category (i.e., 7.5+ MET-h/wk), which was open-ended.

**Table S13.** Sensitivity analysis 2: Joint associations of leisure-time physical activity (using a new categorization) and ambient PM_2.5_ with all-cause and specific-cause mortality in adults using multiple imputation across different subgroup populations (n=869,038)

| Population | PM_2.5_ (μg/m^3^) | LTPA^a^ | All-cause Mortality^b^ | | Cancer Mortality^b, c^ | | Cardiovascular Mortality^b, c^ | |
| --- | --- | --- | --- | --- | --- | --- | --- | --- |
|  |  |  | Death | aHR (95% CI) | Death | aHR (95% CI) | Death | aHR (95% CI) |
| Total |  |  |  |  |  |  |  |  |
| n=869,038 | 35–50 | 0 (METs-hr/wk) | 45,804 | Ref | 22,465 | Ref | 8,771 | Ref |
|  | 35–50 | 2^nd^ & 3^rd^ tertile |  | **0.76 (0.65, 0.89)** |  | 1.00 (0.77, 1.29) |  | **0.58 (0.40, 0.84)** |
|  | 25–35 | 2^nd^ & 3^rd^ tertile |  | **0.69 (0.60, 0.79)** |  | 0.84 (0.67, 1.07) |  | **0.54 (0.39, 0.75)** |
|  | 15–25 | 2^nd^ & 3^rd^ tertile |  | **0.31 (0.27, 0.36)** |  | **0.37 (0.29, 0.47)** |  | **0.31 (0.22, 0.42)** |
|  | 10–15 | 2^nd^ & 3^rd^ tertile |  | **0.33 (0.27, 0.40)** |  | **0.39 (0.28, 0.54)** |  | **0.37 (0.24, 0.57)** |
|  | <10 | 2^nd^ & 3^rd^ tertile |  | **0.30 (0.25, 0.37)** |  | **0.37 (0.27, 0.51)** |  | **0.32 (0.21, 0.50)** |
| Subgroup |  |  |  |  |  |  |  |  |
| Sex |  |  |  |  |  |  |  |  |
| Female | 35–50 | 0 (METs-hr/wk) | 18,704 | Ref | 10,331 | Ref | 2,638 | Ref |
| (n=479,701) | 35–50 | 2^nd^ & 3^rd^ tertile |  | **0.73 (0.58, 0.93)** |  | 1.03 (0.71, 1.49) |  | 0.59 (0.32, 1.09) |
|  | 25–35 | 2^nd^ & 3^rd^ tertile |  | **0.62 (0.50, 0.77)** |  | 0.71 (0.51, 1.00) |  | **0.51 (0.30, 0.86)** |
|  | 15–25 | 2^nd^ & 3^rd^ tertile |  | **0.29 (0.24, 0.37)** |  | **0.34 (0.24, 0.48)** |  | **0.32 (0.19, 0.54)** |
|  | 10–15 | 2^nd^ & 3^rd^ tertile |  | **0.31 (0.23, 0.43)** |  | **0.33 (0.21, 0.53)** |  | **0.48 (0.23, 0.97)** |
|  | <10 | 2^nd^ & 3^rd^ tertile |  | **0.29 (0.21, 0.40)** |  | **0.31 (0.20, 0.49)** |  | **0.43 (0.21, 0.89)** |
| Male | 35–50 | 0 (METs-hr/wk) | 27,100 | Ref | 12,134 | Ref | 6,133 | Ref |
| (n=38,9337) | 35–50 | 2^nd^ & 3^rd^ tertile |  | **0.79 (0.64, 0.98)** |  | 0.99 (0.69, 1.42) |  | **0.58 (0.36, 0.95)** |
|  | 25–35 | 2^nd^ & 3^rd^ tertile |  | **0.75 (0.62, 0.91)** |  | 0.97 (0.70, 1.34) |  | **0.59 (0.39, 0.89)** |
|  | 15–25 | 2^nd^ & 3^rd^ tertile |  | **0.32 (0.27, 0.39)** |  | **0.41 (0.29, 0.57)** |  | **0.31 (0.21, 0.47)** |
|  | 10–15 | 2^nd^ & 3^rd^ tertile |  | **0.35 (0.27, 0.45)** |  | **0.44 (0.29, 0.67)** |  | **0.34 (0.20, 0.59)** |
|  | <10 | 2^nd^ & 3^rd^ tertile |  | **0.31 (0.24, 0.41)** |  | **0.42 (0.27, 0.65)** |  | **0.30 (0.17, 0.52)** |
| Age |  |  |  |  |  |  |  |  |
| 65+ | 35–50 | 0 (METs-hr/wk) | 18,586 | Ref | 8,252 | Ref | 3,857 | Ref |
| (n=118,129) | 35–50 | 2^nd^ & 3^rd^ tertile |  | **0.71 (0.56, 0.91)** |  | 1.03 (0.67, 1.60) |  | **0.56 (0.32, 0.97)** |
|  | 25–35 | 2^nd^ & 3^rd^ tertile |  | **0.62 (0.50, 0.78)** |  | 0.77 (0.52, 1.15) |  | **0.51 (0.32, 0.83)** |
|  | 15–25 | 2^nd^ & 3^rd^ tertile |  | **0.29 (0.23, 0.36)** |  | **0.32 (0.21, 0.48)** |  | **0.32 (0.20, 0.52)** |
|  | 10–15 | 2^nd^ & 3^rd^ tertile |  | **0.31 (0.22, 0.43)** |  | **0.37 (0.21, 0.62)** |  | **0.32 (0.16, 0.67)** |
|  | <10 | 2^nd^ & 3^rd^ tertile |  | **0.29 (0.21, 0.40)** |  | **0.36 (0.21, 0.61)** |  | **0.29 (0.14, 0.61)** |
| 18-64 | 35–50 | 0 (METs-hr/wk) | 27,218 | Ref | 14,213 | Ref | 4,914 | Ref |
| (n=750,909) | 35–50 | 2^nd^ & 3^rd^ tertile |  | **0.70 (0.57, 0.87)** |  | 0.90 (0.65, 1.23) |  | **0.51 (0.30, 0.85)** |
|  | 25–35 | 2^nd^ & 3^rd^ tertile |  | **0.67 (0.56, 0.81)** |  | 0.82 (0.61, 1.09) |  | **0.51 (0.33, 0.80)** |
|  | 15–25 | 2^nd^ & 3^rd^ tertile |  | **0.27 (0.22, 0.32)** |  | **0.33 (0.25, 0.45)** |  | **0.24 (0.16, 0.38)** |
|  | 10–15 | 2^nd^ & 3^rd^ tertile |  | **0.31 (0.25, 0.40)** |  | **0.37 (0.25, 0.55)** |  | **0.35 (0.20, 0.61)** |
|  | <10 | 2^nd^ & 3^rd^ tertile |  | **0.30 (0.24, 0.39)** |  | **0.37 (0.25, 0.55)** |  | **0.32 (0.19, 0.57)** |
| CVDs |  |  |  |  |  |  |  |  |
| Yes | 35–50 | 0 (METs-hr/wk) | 21,026 | Ref | 8,766 | Ref | 5,341 | Ref |
| (n=193,769) | 35–50 | 2^nd^ & 3^rd^ tertile |  | 0.75 (0.55, 1.03) |  | 0.97 (0.57, 1.66) |  | 0.62 (0.34, 1.12) |
|  | 25–35 | 2^nd^ & 3^rd^ tertile |  | **0.63 (0.47, 0.83)** |  | 0.68 (0.42, 1.13) |  | **0.47 (0.27, 0.80)** |
|  | 15–25 | 2^nd^ & 3^rd^ tertile |  | **0.30 (0.23, 0.40)** |  | **0.32 (0.20, 0.53)** |  | **0.26 (0.15, 0.45)** |
|  | 10–15 | 2^nd^ & 3^rd^ tertile |  | **0.32 (0.23, 0.46)** |  | **0.30 (0.17, 0.55)** |  | **0.35 (0.18, 0.67)** |
|  | <10 | 2^nd^ & 3^rd^ tertile |  | **0.29 (0.21, 0.42)** |  | **0.29 (0.16, 0.52)** |  | **0.31 (0.16, 0.58)** |
| No | 35–50 | 0 (METs-hr/wk) | 24,778 | Ref | 13,699 | Ref | 3,430 | Ref |
| (n=675,269) | 35–50 | 2^nd^ & 3^rd^ tertile |  | **0.76 (0.63, 0.91)** |  | 1.01 (0.75, 1.35) |  | **0.52 (0.32, 0.86)** |
|  | 25–35 | 2^nd^ & 3^rd^ tertile |  | **0.72 (0.61, 0.85)** |  | 0.92 (0.70, 1.20) |  | **0.60 (0.40, 0.91)** |
|  | 15–25 | 2^nd^ & 3^rd^ tertile |  | **0.31 (0.27, 0.37)** |  | **0.40 (0.30, 0.52)** |  | **0.35 (0.23, 0.52)** |
|  | 10–15 | 2^nd^ & 3^rd^ tertile |  | **0.34 (0.26, 0.43)** |  | **0.44 (0.30, 0.64)** |  | **0.36 (0.19, 0.66)** |
|  | <10 | 2^nd^ & 3^rd^ tertile |  | **0.31 (0.24, 0.39)** |  | **0.42 (0.29, 0.61)** |  | **0.32 (0.17, 0.59)** |

aHR: adjusted hazard ratio; CI: confidence interval; CVDs: cardiovascular diseases; LTPA, leisure-time physical activity; MET-h, metabolic equivalent hours; PM_2.5_: fine particulate matter 2.5

Bold values indicate statistical significance.

a: Participants who did not engage in any LTPA were used as the reference group. The remaining participants, who were active during leisure time, were categorized into tertiles based on their activity levels, measured in METs-hours per week. The 2^nd^ & 3^rd^ tertiles of LTPA levels within each cohort: MJ cohort (> percentile rank 33: 1.25+); Taiwan Biobank ((> percentile rank 33: 14.40+), and UK Biobank ((> percentile rank 33: 4.78+).

b: Adjusted for sex, age, educational levels, household income, smoking, alcohol consumption, body mass index, number of chronic diseases, and cohort.

c: Using cause-specific hazard models for competing risks of other mortality causes.

The category ranges of PM_2.5_ were defined as lower bound inclusive and upper bound exclusive.

**Table S14**. Sensitivity analysis 3: Joint associations of leisure-time physical activity and ambient PM_2.5_ with all-cause and cause-specific mortality based on complete-case analysis (n=713,120).

| Population | PM_2.5_ (μg/m^3^) | LTPA  (MET-h/week) | All-cause Mortality^a^ | | Cancer Mortality^a, b^ | | Cardiovascular Mortality^a, b^ | |
| --- | --- | --- | --- | --- | --- | --- | --- | --- |
|  |  |  | Death | aHR (95% CI) | Death | aHR (95% CI) | Death | aHR (95% CI) |
| Total |  |  |  |  |  |  |  |  |
| n=713,120 | 35-50 | <1 | 32,771 | Ref | 16,459 | Ref | 6,213 | Ref |
|  | 35-50 | 7.5+ |  | **0.72 (0.57, 0.91)** |  | 1.07 (0.72, 1.58) |  | **0.58 (0.34, 0.99)** |
|  | 25-35 | 7.5+ |  | **0.59 (0.48, 0.73)** |  | 0.85 (0.59, 1.22) |  | **0.44 (0.27, 0.69)** |
|  | 15-25 | 7.5+ |  | **0.29 (0.24, 0.36)** |  | **0.39 (0.27, 0.56)** |  | **0.28 (0.18, 0.44)** |
|  | 10-15 | 7.5+ |  | **0.28 (0.22, 0.36)** |  | **0.38 (0.25, 0.58)** |  | **0.32 (0.19, 0.56)** |
|  | <10 | 7.5+ |  | **0.26 (0.20, 0.34)** |  | **0.36 (0.23, 0.55)** |  | **0.29 (0.17, 0.50)** |
| Subgroup |  |  |  |  |  |  |  |  |
| Sex |  |  |  |  |  |  |  |  |
| Female | 35-50 | <1 | 12,741 | Ref | 7,275 | Ref | 1,750 | Ref |
| (n=387,530) | 35-50 | 7.5+ |  | 0.73 (0.51, 1.03) |  | 0.98 (0.57, 1.69) |  | 0.64 (0.29, 1.40) |
|  | 25-35 | 7.5+ |  | **0.54 (0.40, 0.73)** |  | 0.63 (0.38, 1.04) |  | **0.34 (0.17, 0.67)** |
|  | 15-25 | 7.5+ |  | **0.28 (0.20, 0.38)** |  | **0.32 (0.20, 0.53)** |  | **0.24 (0.12, 0.47)** |
|  | 10-15 | 7.5+ |  | **0.26 (0.18, 0.39)** |  | **0.27 (0.14, 0.50)** |  | **0.40 (0.17, 0.92)** |
|  | <10 | 7.5+ |  | **0.25 (0.17, 0.37)** |  | **0.26 (0.14, 0.48)** |  | **0.35 (0.15, 0.82)** |
| Male | 35-50 | <1 | 20,030 | Ref | 9,184 | Ref | 4,463 | Ref |
| (n=325,590) | 35-50 | 7.5+ |  | 0.73 (0.54, 1.00) |  | 1.20 (0.68, 2.10) |  | 0.61 (0.30, 1.24) |
|  | 25-35 | 7.5+ |  | **0.64 (0.49, 0.85)** |  | 1.10 (0.65, 1.86) |  | 0.56 (0.30, 1.04) |
|  | 15-25 | 7.5+ |  | **0.30 (0.23, 0.40)** |  | **0.48 (0.28, 0.80)** |  | **0.34 (0.18, 0.63)** |
|  | 10-15 | 7.5+ |  | **0.30 (0.21, 0.42)** |  | **0.49 (0.27, 0.89)** |  | **0.33 (0.16, 0.69)** |
|  | <10 | 7.5+ |  | **0.27 (0.19, 0.38)** |  | **0.46 (0.25, 0.84)** |  | **0.30 (0.14, 0.62)** |
| Age |  |  |  |  |  |  |  |  |
| 65+ | 35-50 | <1 | 12,763 | Ref | 5,792 | Ref | 2,637 | Ref |
| (n=87,236) | 35-50 | 7.5+ |  | 0.80 (0.55, 1.16) |  | 1.79 (0.81, 3.97) |  | 0.80 (0.34, 1.86) |
|  | 25-35 | 7.5+ |  | **0.69 (0.49, 0.97)** |  | 1.21 (0.57, 2.58) |  | 0.61 (0.28, 1.33) |
|  | 15-25 | 7.5+ |  | **0.32 (0.23, 0.45)** |  | 0.54 (0.25, 1.14) |  | **0.39 (0.18, 0.85)** |
|  | 10-15 | 7.5+ |  | **0.31 (0.20, 0.48)** |  | 0.52 (0.22, 1.24) |  | 0.42 (0.16, 1.12) |
|  | <10 | 7.5+ |  | **0.29 (0.18, 0.45)** |  | 0.51 (0.21, 1.20) |  | 0.38 (0.14, 1.01) |
| 18-64 | 35-50 | <1 | 20,008 | Ref | 10,667 | Ref | 3,576 | Ref |
| (n=625,884) | 35-50 | 7.5+ |  | **0.68 (0.51, 0.92)** |  | 0.89 (0.56, 1.40) |  | **0.45 (0.22, 0.90)** |
|  | 25-35 | 7.5+ |  | **0.59 (0.45, 0.76)** |  | 0.84 (0.56, 1.27) |  | **0.38 (0.21, 0.68)** |
|  | 15-25 | 7.5+ |  | **0.28 (0.21, 0.36)** |  | **0.37 (0.24, 0.55)** |  | **0.23 (0.13, 0.40)** |
|  | 10-<15 | 7.5+ |  | **0.25 (0.18, 0.34)** |  | **0.32 (0.20, 0.53)** |  | **0.26 (0.13, 0.50)** |
|  | <10 | 7.5+ |  | **0.25 (0.18, 0.34)** |  | **0.33 (0.20, 0.54)** |  | **0.24 (0.12, 0.48)** |
| CVDs |  |  |  |  |  |  |  |  |
| Yes | 35-50 | <1 | 14,735 | Ref | 6,281 | Ref | 3,710 | Ref |
| (n=151,989) | 35-50 | 7.5+ |  | 0.80 (0.54, 1.19) |  | 1.11 (0.55, 2.21) |  | 0.59 (0.29, 1.19) |
|  | 25-35 | 7.5+ |  | **0.53 (0.37, 0.76)** |  | 0.62 (0.33, 1.19) |  | **0.29 (0.15, 0.56)** |
|  | 15-25 | 7.5+ |  | **0.28 (0.19, 0.40)** |  | **0.30 (0.16, 0.58)** |  | **0.20 (0.11, 0.38)** |
|  | 10-15 | 7.5+ |  | **0.28 (0.18, 0.42)** |  | **0.27 (0.13, 0.56)** |  | **0.26 (0.13, 0.55)** |
|  | <10 | 7.5+ |  | **0.25 (0.17, 0.38)** |  | **0.25 (0.12, 0.53)** |  | **0.22 (0.11, 0.47)** |
| No | 35-50 | <1 | 18,036 | Ref | 10,178 | Ref | 2,503 | Ref |
| (n=561,131) | 35-50 | 7.5+ |  | **0.67 (0.50, 0.89)** |  | 1.05 (0.65, 1.69) |  | 0.48 (0.22, 1.08) |
|  | 25-35 | 7.5+ |  | **0.63 (0.49, 0.82)** |  | 0.99 (0.64, 1.53) |  | 0.60 (0.31, 1.17) |
|  | 15-25 | 7.5+ |  | **0.30 (0.23, 0.38)** |  | **0.45 (0.29, 0.69)** |  | **0.36 (0.19, 0.69)** |
|  | 10-15 | 7.5+ |  | **0.28 (0.20, 0.39)** |  | **0.46 (0.27, 0.77)** |  | **0.34 (0.15, 0.79)** |
|  | <10 | 7.5+ |  | **0.26 (0.19, 0.37)** |  | **0.44 (0.26, 0.73)** |  | **0.32 (0.14, 0.74)** |

aHR: adjusted hazard ratio; CI: confidence interval; CVDs: cardiovascular diseases; LTPA, leisure-time physical activity; MET-h, metabolic equivalent hours; PM_2.5_: fine particulate matter 2.5

Bold values indicate statistical significance.

a: Adjusted for sex, age, educational levels, household income, smoking, alcohol consumption, body mass index, number of chronic diseases, and cohort.

b: Using cause-specific hazard models for competing risks of other mortality causes.

The category ranges of PM_2.5_ and LTPA were defined as lower bound inclusive and upper bound exclusive, except for the highest category (i.e., 7.5+ MET-h/wk), which was open-ended.

**Table S15.** Multiplicative and additive interactions between leisure-time physical activity and ambient PM_2.5_ on all-cause mortality (n= 869,038)

| **Indicators** | **Interaction** | | | | |
| --- | --- | --- | --- | --- | --- |
|  | **Multiplicative** | | **Additive** (95% CI) ^b^ | | |
| **Effect size** |  |  | | | |
| Overall Wald test | χ^2^ = 82.8 | | - | | |
| df | 12 | | - | | |
| p-value | <0.001 | | - | | |
| **(PM_2.5_ x LTPA) group** ^a^ |  | | **(35-50) x (<1) as reference group** | | |
|  |  |  | **RERI** | **AP** | **SI** |
| (25-35) x (1-7.5) | - | | 0.446 (0.169, 0.721) | 1.180 (0.886, 1.473) | 0.581 (0.458, 0.739) |
| (25-35) x (7.5-15) | - | | 0.428 (-0.012, 0.869) | 0.671 (0.492, 0.850) | 0.457 (0.341, 0.612) |
| (25-35) x (15+) | - | | 0.361 (-0.053, 0.777) | 0.647 (0.522, 0.772) | 0.549 (0.410, 0.734) |
| (15-25) x (1-7.5) | - | | 0.500 (0.345, 0.654) | 4.170 (4.015, 4.325) | 0.637 (0.502, 0.808) |
| (15-25) x (7.5-15) | - | | 0.361 (0.082, 0.641) | 1.393 (1.065, 1.722) | 0.671 (0.523, 0.896) |
| (15-25) x (15+) | - | | 0.346 (0.077, 0.616) | 1.495 (1.254, 1.736) | 0.688 (0.516, 0.919) |
| (10-15) x (1-7.5) | - | | 0.265 (-0.133, 0.665) | 0.426 (0.238, 0.613) | 0.585 (0.469, 0.730) |
| (10-15) x (7.5-15) | - | | -0.070 (-0.550, 0.410) | -0.123 (-0.490, 0.242) | 1.192 (0.913, 1.557) |
| (10-15) x (15+) | - | | -0.067 (-0.115, 0.407) | -0.121 (-0.488, 0.244) | 1.179 (0.903, 1.541) |
| (<10) x (1-7.5) | - | | 0.269 (-0.115, 0.653) | 0.459 (0.278, 0.639) | 0.605 (0.485, 0.755) |
| (<10) x (7.5-15) | - | | -0.072 (-0.534, 0.390) | -0.138 (-0.539, 0.263) | 1.178 (0.902, 1.538) |
| (<10) x (15+) | - | | -0.060 (-0.520, 0.398) | -0.116 (-0.503, 0.270) | 1.145 (0.876, 1.496) |

aHR: adjusted hazard ratio; CI: confidence interval; LTPA: leisure-time physical activity; MET-h: metabolic equivalent hours; PM_2.5_: fine particulate matter 2.5.

a: Unit of PM_2.5_: μg/m^3^; LTPA: MET-h/wk.

b: Indicators of additive interaction: RERI, relative excess risk due to interaction (RR_11_−RR_10_−RR_01_+1); AP, proportion attributable to interaction (RERI/RR_11_); SI, synergy index ([RR_11_–1]/[(RR_10_–1)+(RR_01_–1)]).

**Table S16.** Numbers of participants and deaths across categories of PM_2.5_ exposure and leisure-time physical activity in the UK and Taiwan cohorts (n = 869,038)

| n (%) | LTPA (MET-h/week) | | | |
| --- | --- | --- | --- | --- |
|  | <1 | 1-7.5 | 7.5-15 | 15+ |
| PM_2.5_ (μg/m^3^) |  |  |  |  |
| 35-50 | 2,123 (0.24) | 9,128 (1.05) | 1,668 (0.19) | 1,664 (0.19) |
| Participant / Death |  |  |  |  |
| UK | 104 / 16 | 33 / 4 | 8 / 1 | 4 / 0 |
| Taiwan | 2,019 / 217 | 9,095 / 499 | 1,660 / 154 | 1,660 / 163 |
| 25-35 | 16,484 (1.90) | 36,362 (4.18) | 7,752 (0.89) | 10,818 (1.24) |
| Participant / Death |  |  |  |  |
| UK | 12 / 0 | 6 / 0 | 0 / 0 | 0 / 0 |
| Taiwan | 16,472 / 800 | 36,356 / 1,546 | 7,752 / 486 | 10,818 / 550 |
| 15-25 | 77,042 (8.87) | 111,640 (12.85) | 25,207 (2.9) | 36,704 (4.22) |
| Participant / Death |  |  |  |  |
| UK | 117 / 18 | 105 / 5 | 69 / 3 | 98 / 6 |
| Taiwan | 76,925 / 825 | 111,535 / 1,448 | 25,138 / 523 | 36,606 / 545 |
| 10-15 | 77,657 (8.94) | 74,471 (8.57) | 46,654 (5.37) | 64,859 (7.46) |
| Participant / Death |  |  |  |  |
| UK | 61,113 / 7,151 | 73,249 / 5,194 | 44,011 / 2,892 | 57,460 / 3,813 |
| Taiwan | 16,544 / 62 | 1,222 / 7 | 2,643 / 23 | 7,399 / 53 |
| <10 | 56,584 (6.51) | 82,373 (9.48) | 53,887 (6.20) | 75,961 (8.74) |
| Participant / Death |  |  |  |  |
| UK | 54,198 / 5,786 | 82,209 / 5,366 | 53,461 / 3,161 | 74,831 / 4,476 |
| Taiwan | 2,386 / 6 | 164 / 0 | 426 / 1 | 1,130 / 4 |

LTPA, leisure-time physical activity; MET-h, metabolic equivalent hours; PM_2.5_: fine particulate matter 2.5

The category ranges of PM_2.5_ and LTPA were defined as lower bound inclusive and upper bound exclusive, except for the highest category (i.e., 15+ MET-h/wk), which was open-ended.


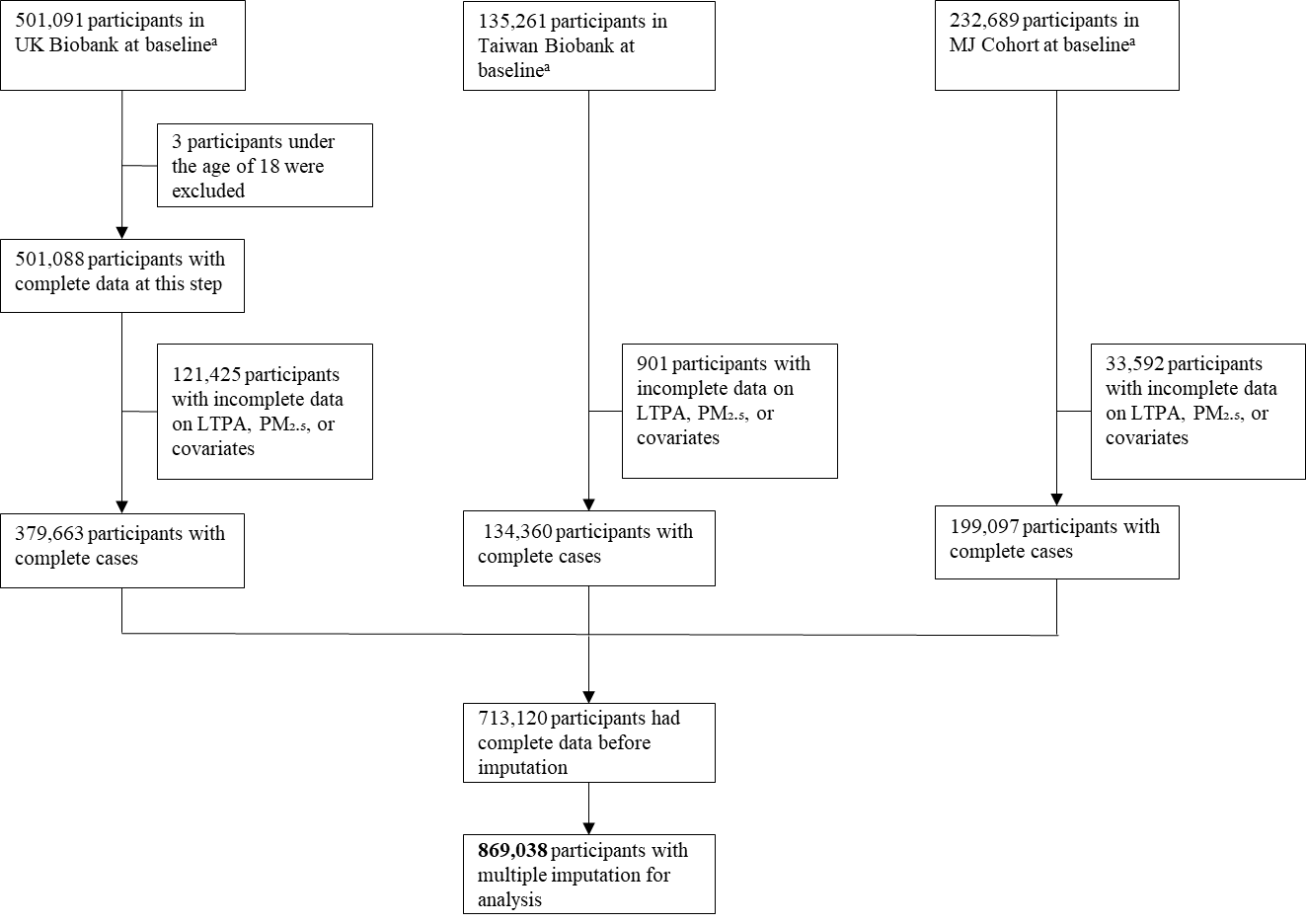


**Figure S1.** The flowchart of the analytical sample selection in the pooled individual participant data analysis


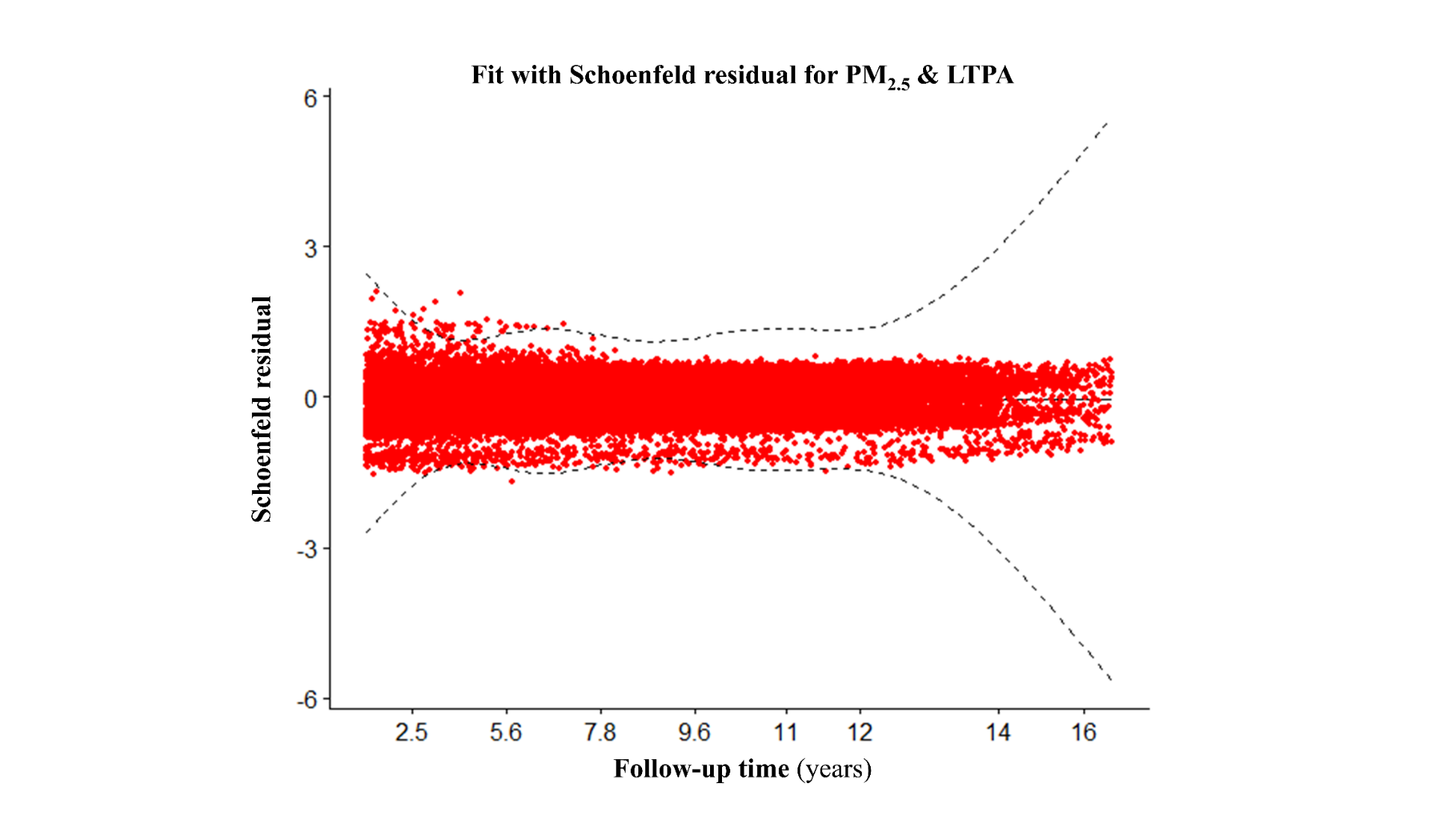


**Figure S2.** Test for Cox proportional hazards using scaled Schoenfeld residual

Flat lines at 0 suggest that the coefficient does not vary over time and that proportional hazards hold

**
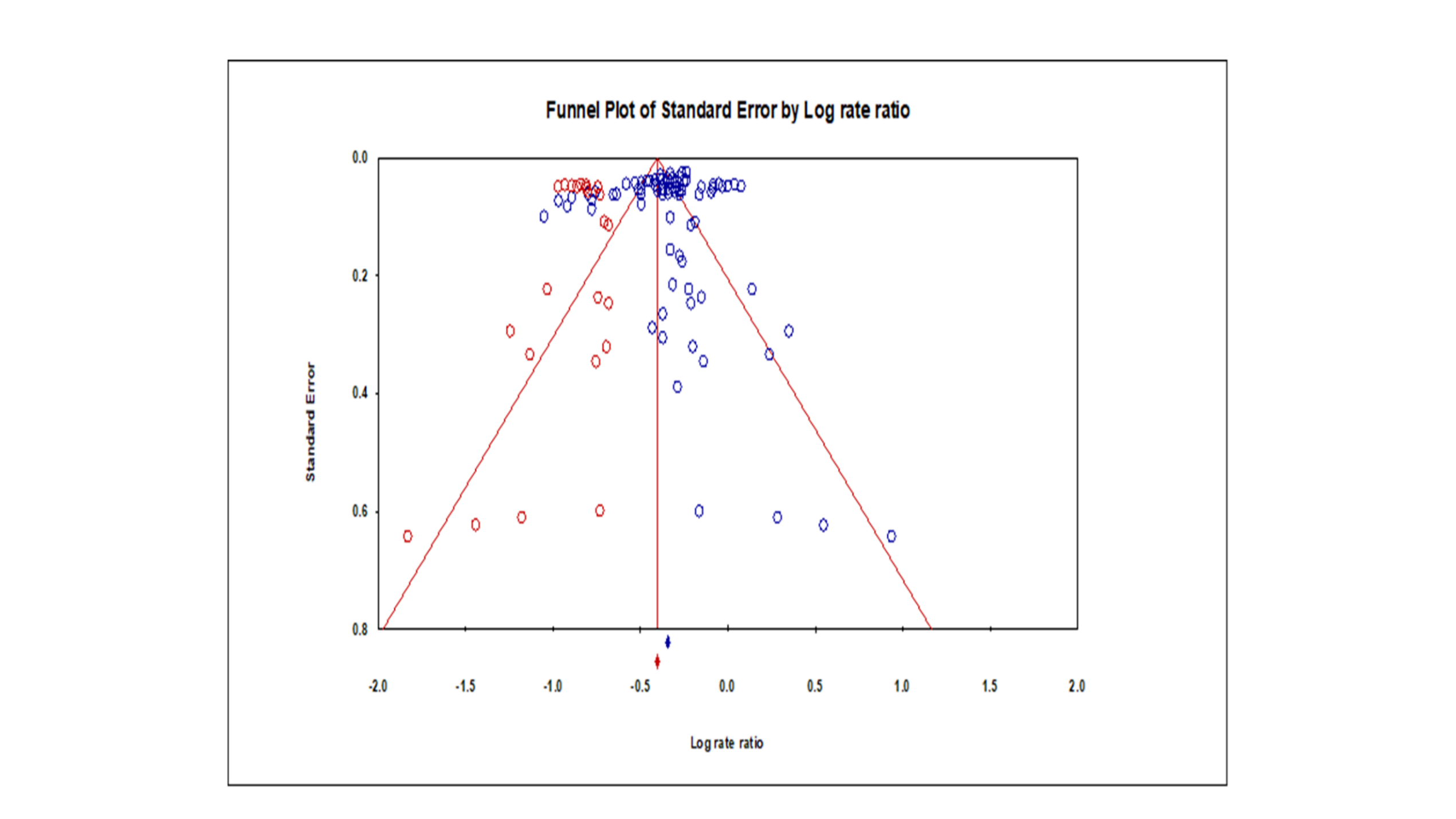
**

**Figure S3.** Funnel plot with imputed studies using random effects model

Blue circles: effect sizes based on original studies; red circles: imputed effect sizes


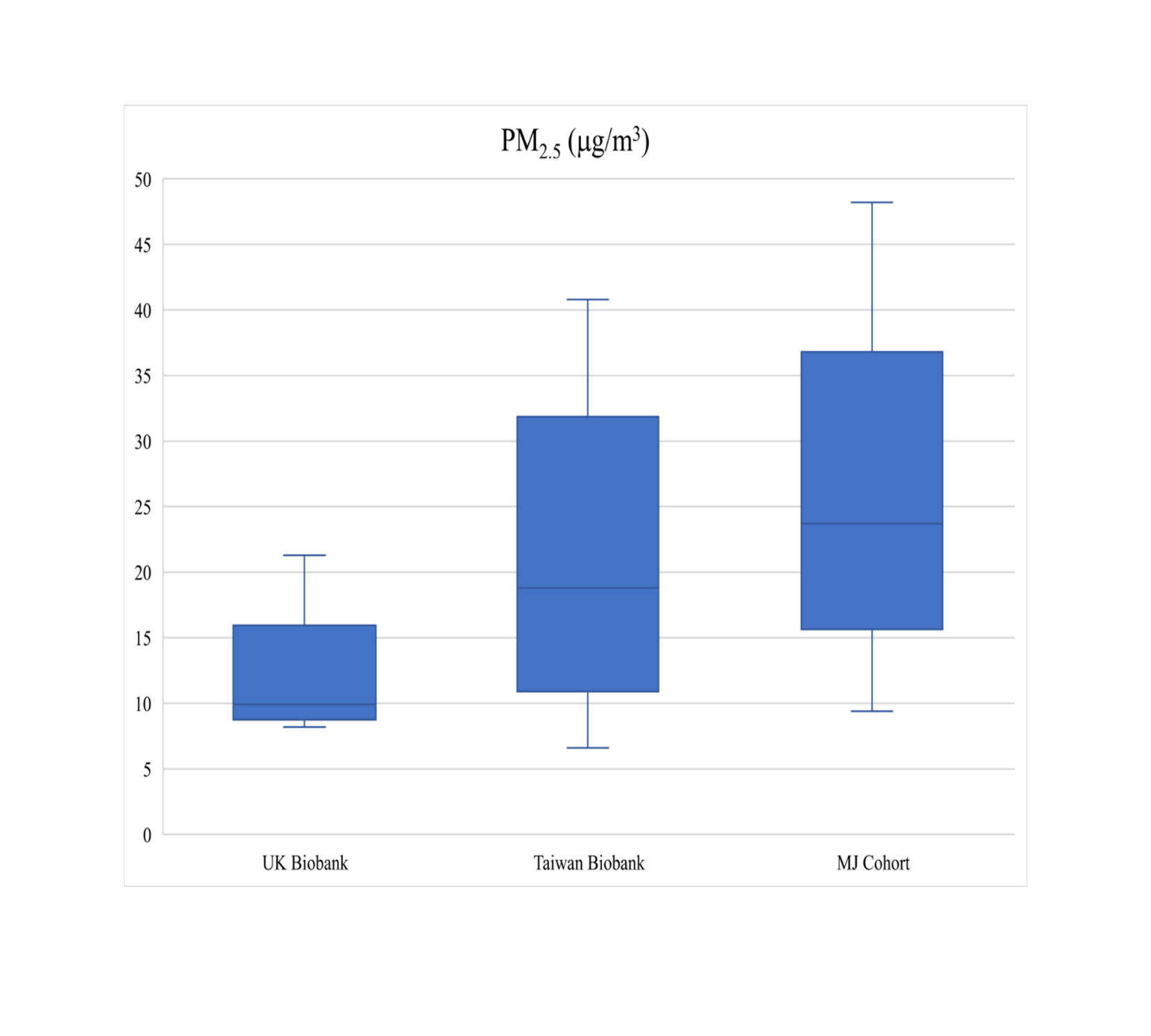


**Figure S4.** Annual average distribution of ambient PM_2.5_ concentrations in the included cohort data during follow-up periods using box plots

The box plots summarize each cohort's data, presenting the maximum, third quartile, median, first quartile, and minimum values from top to bottom.

| **Section and Topic**  PRISMA 2020 | **Item #** | **Checklist item** | **Location where item is reported** |
| --- | --- | --- | --- |
| **TITLE** | | |  |
| Title | 1 | Identify the report as a systematic review. | Title page, P.1 |
| **ABSTRACT** | | |  |
| Abstract | 2 | See the PRISMA 2020 for Abstracts checklist. | Abstract; N.A. (This manuscript includes two studies) |
| **INTRODUCTION** | | |  |
| Rationale | 3 | Describe the rationale for the review in the context of existing knowledge. | PP.3-4 |
| Objectives | 4 | Provide an explicit statement of the objective(s) or question(s) the review addresses. | P.4 |
| **METHODS** | | |  |
| Eligibility criteria | 5 | Specify the inclusion and exclusion criteria for the review and how studies were grouped for the syntheses. | P.5 |
| Information sources | 6 | Specify all databases, registers, websites, organisations, reference lists and other sources searched or consulted to identify studies. Specify the date when each source was last searched or consulted. | P.4 |
| Search strategy | 7 | Present the full search strategies for all databases, registers and websites, including any filters and limits used. | PP.4-5 |
| Selection process | 8 | Specify the methods used to decide whether a study met the inclusion criteria of the review, including how many reviewers screened each record and each report retrieved, whether they worked independently, and if applicable, details of automation tools used in the process. | PP.5-6 |
| Data collection process | 9 | Specify the methods used to collect data from reports, including how many reviewers collected data from each report, whether they worked independently, any processes for obtaining or confirming data from study investigators, and if applicable, details of automation tools used in the process. | P.6 |
| Data items | 10a | List and define all outcomes for which data were sought. Specify whether all results that were compatible with each outcome domain in each study were sought (e.g. for all measures, time points, analyses), and if not, the methods used to decide which results to collect. | P.6 |
|  | 10b | List and define all other variables for which data were sought (e.g. participant and intervention characteristics, funding sources). Describe any assumptions made about any missing or unclear information. | PP.6-7; Table S6 |
| Study risk of bias assessment | 11 | Specify the methods used to assess risk of bias in the included studies, including details of the tool(s) used, how many reviewers assessed each study and whether they worked independently, and if applicable, details of automation tools used in the process. | P.6 |
| Effect measures | 12 | Specify for each outcome the effect measure(s) (e.g. risk ratio, mean difference) used in the synthesis or presentation of results. | PP.6-7; Table S6 |
| Synthesis methods | 13a | Describe the processes used to decide which studies were eligible for each synthesis (e.g. tabulating the study intervention characteristics and comparing against the planned groups for each synthesis (item #5)). | Figure 1 & Table A6 |
|  | 13b | Describe any methods required to prepare the data for presentation or synthesis, such as handling of missing summary statistics, or data conversions. | pp.6-7, Table S2 |
|  | 13c | Describe any methods used to tabulate or visually display results of individual studies and syntheses. | P.8 |
|  | 13d | Describe any methods used to synthesize results and provide a rationale for the choice(s). If meta-analysis was performed, describe the model(s), method(s) to identify the presence and extent of statistical heterogeneity, and software package(s) used. | PP.7-8 |
|  | 13e | Describe any methods used to explore possible causes of heterogeneity among study results (e.g. subgroup analysis, meta-regression). | PP.7-8 |
|  | 13f | Describe any sensitivity analyses conducted to assess robustness of the synthesized results. | P.8 |
| Reporting bias assessment | 14 | Describe any methods used to assess risk of bias due to missing results in a synthesis (arising from reporting biases). | P.8-9 |
| Certainty assessment | 15 | Describe any methods used to assess certainty (or confidence) in the body of evidence for an outcome. | P.9 |
| **RESULTS** | | |  |
| Study selection | 16a | Describe the results of the search and selection process, from the number of records identified in the search to the number of studies included in the review, ideally using a flow diagram. | P.13 & Fig. 1 |
|  | 16b | Cite studies that might appear to meet the inclusion criteria, but which were excluded, and explain why they were excluded. | P.13 & Fig. 1 |
| Study characteristics | 17 | Cite each included study and present its characteristics. | P.13, Table 1 & Table S6 |
| Risk of bias in studies | 18 | Present assessments of risk of bias for each included study. | P.13 & Table S7 |
| Results of individual studies | 19 | For all outcomes, present, for each study: (a) summary statistics for each group (where appropriate) and (b) an effect estimate and its precision (e.g. confidence/credible interval), ideally using structured tables or plots. | Table S6 |
| Results of syntheses | 20a | For each synthesis, briefly summarise the characteristics and risk of bias among contributing studies. | P.13 & Table S7 |
|  | 20b | Present results of all statistical syntheses conducted. If meta-analysis was done, present for each the summary estimate and its precision (e.g. confidence/credible interval) and measures of statistical heterogeneity. If comparing groups, describe the direction of the effect. | PP.16-18 |
|  | 20c | Present results of all investigations of possible causes of heterogeneity among study results. | P.19, Table S8 |
|  | 20d | Present results of all sensitivity analyses conducted to assess the robustness of the synthesized results. | P.19, Table S8 |
| Reporting biases | 21 | Present assessments of risk of bias due to missing results (arising from reporting biases) for each synthesis assessed. | P.19 & Fig. S3 |
| Certainty of evidence | 22 | Present assessments of certainty (or confidence) in the body of evidence for each outcome assessed. | P.20 & Table S9 |
| **DISCUSSION** | | |  |
| Discussion | 23a | Provide a general interpretation of the results in the context of other evidence. | P.30 |
|  | 23b | Discuss any limitations of the evidence included in the review. | PP.33-34 |
|  | 23c | Discuss any limitations of the review processes used. | PP.33-34 |
|  | 23d | Discuss implications of the results for practice, policy, and future research. | PP.32-33 |
| **OTHER INFORMATION** | | |  |
| Registration and protocol | 24a | Provide registration information for the review, including register name and registration number, or state that the review was not registered. | Abstract & P.2 |
|  | 24b | Indicate where the review protocol can be accessed, or state that a protocol was not prepared. | P.2 |
|  | 24c | Describe and explain any amendments to information provided at registration or in the protocol. | P.2 |
| Support | 25 | Describe sources of financial or non-financial support for the review, and the role of the funders or sponsors in the review. | P.36 |
| Competing interests | 26 | Declare any competing interests of review authors. | P.36 |
| Availability of data, code and other materials | 27 | Report which of the following are publicly available and where they can be found: template data collection forms; data extracted from included studies; data used for all analyses; analytic code; any other materials used in the review. | P.37 |

*From:*  Page MJ, McKenzie JE, Bossuyt PM, Boutron I, Hoffmann TC, Mulrow CD, et al. The PRISMA 2020 statement: an updated guideline for reporting systematic reviews. BMJ 2021;372:n71. doi: 10.1136/bmj.n71 For more information, visit: <http://www.prisma-statement.org/>

STROBE Statement—checklist of items that should be included in reports of observational studies

|  | | **Item No** | **Recommendation** | **Page  No** |  |
| --- | --- | --- | --- | --- | --- |
| **Title and abstract** | | 1 | (*a*) Indicate the study’s design with a commonly used term in the title or the abstract | 1 |  |
|  |  |  | (*b*) Provide in the abstract an informative and balanced summary of what was done and what was found | Abstract |  |
| **Introduction** | | | | |  |
| Background/rationale | | 2 | Explain the scientific background and rationale for the investigation being reported | 3-4 |  |
| Objectives | | 3 | State specific objectives, including any prespecified hypotheses | 4 |  |
| **Methods** | | | | |  |
| Study design | | 4 | Present key elements of study design early in the paper | 9-10 |  |
| Setting | | 5 | Describe the setting, locations, and relevant dates, including periods of recruitment, exposure, follow-up, and data collection | 9-10; Table S6 & Figure S1 |  |
| Participants | | 6 | (*a*) *Cohort study*—Give the eligibility criteria, and the sources and methods of selection of participants. Describe methods of follow-up  *Case-control study*—Give the eligibility criteria, and the sources and methods of case ascertainment and control selection. Give the rationale for the choice of cases and controls  *Cross-sectional study*—Give the eligibility criteria, and the sources and methods of selection of participants | 9-10 & Figure S1 |  |
|  |  |  | (*b*) *Cohort study*—For matched studies, give matching criteria and number of exposed and unexposed  *Case-control study*—For matched studies, give matching criteria and the number of controls per case |  |  |
| Variables | | 7 | Clearly define all outcomes, exposures, predictors, potential confounders, and effect modifiers. Give diagnostic criteria, if applicable | 10-11 |  |
| Data sources/ measurement | | 8* | For each variable of interest, give sources of data and details of methods of assessment (measurement). Describe comparability of assessment methods if there is more than one group | Table S4; Table 2 |  |
| Bias | | 9 | Describe any efforts to address potential sources of bias | 12 |  |
| Study size | | 10 | Explain how the study size was arrived at | 9-10; Figure. S1 |  |
| Quantitative variables | | 11 | Explain how quantitative variables were handled in the analyses. If applicable, describe which groupings were chosen and why | 10-11; Table S4 |  |
| Statistical methods | | 12 | (*a*) Describe all statistical methods, including those used to control for confounding | 11-12 |  |
|  |  |  | (*b*) Describe any methods used to examine subgroups and interactions | 11-12 |  |
|  |  |  | (*c*) Explain how missing data were addressed | 12-13 |  |
|  |  |  | (*d*) *Cohort study*—If applicable, explain how loss to follow-up was addressed  *Case-control study*—If applicable, explain how matching of cases and controls was addressed  *Cross-sectional study*—If applicable, describe analytical methods taking account of sampling strategy | 12-13 |  |
|  |  |  | (*e*) Describe any sensitivity analyses | 12-13 |  |
| **Results** | | | | | |
| Participants | 13* | (a) Report numbers of individuals at each stage of study—eg numbers potentially eligible, examined for eligibility, confirmed eligible, included in the study, completing follow-up, and analysed | | 20 | |
|  |  | (b) Give reasons for non-participation at each stage | | Figure S1 | |
|  |  | (c) Consider use of a flow diagram | | Figure S1 | |
| Descriptive data | 14* | (a) Give characteristics of study participants (eg demographic, clinical, social) and information on exposures and potential confounders | | 20; Table 2 | |
|  |  | (b) Indicate number of participants with missing data for each variable of interest | | Figure S1 | |
|  |  | (c) *Cohort study*—Summarise follow-up time (eg, average and total amount) | | 20 | |
| Outcome data | 15* | *Cohort study*—Report numbers of outcome events or summary measures over time | | Table 2 | |
|  |  | *Case-control study—*Report numbers in each exposure category, or summary measures of exposure | |  | |
|  |  | *Cross-sectional study—*Report numbers of outcome events or summary measures | |  | |
| Main results | 16 | (*a*) Give unadjusted estimates and, if applicable, confounder-adjusted estimates and their precision (eg, 95% confidence interval). Make clear which confounders were adjusted for and why they were included | | 22-23; Table S10 to S14 | |
|  |  | (*b*) Report category boundaries when continuous variables were categorized | | Table S4; Table 2 | |
|  |  | (*c*) If relevant, consider translating estimates of relative risk into absolute risk for a meaningful time period | |  | |
| Other analyses | 17 | Report other analyses done—eg analyses of subgroups and interactions, and sensitivity analyses | | 25-26; Figure 5 and Table 3 | |
| **Discussion** | | | | | |
| Key results | 18 | Summarise key results with reference to study objectives | | 30 | |
| Limitations | 19 | Discuss limitations of the study, taking into account sources of potential bias or imprecision. Discuss both direction and magnitude of any potential bias | | 33-34 | |
| Interpretation | 20 | Give a cautious overall interpretation of results considering objectives, limitations, multiplicity of analyses, results from similar studies, and other relevant evidence | | 34-35 | |
| Generalisability | 21 | Discuss the generalisability (external validity) of the study results | | 32-33 | |
| **Other information** | | | | | |
| Funding | 22 | Give the source of funding and the role of the funders for the present study and, if applicable, for the original study on which the present article is based | | 36 | |

*Give information separately for cases and controls in case-control studies and, if applicable, for exposed and unexposed groups in cohort and cross-sectional studies.

**Note:** An Explanation and Elaboration article discusses each checklist item and gives methodological background and published examples of transparent reporting. The STROBE checklist is best used in conjunction with this article (freely available on the Web sites of PLOS Medicine at http://www.plosmedicine.org/, Annals of Internal Medicine at http://www.annals.org/, and Epidemiology at http://www.epidem.com/). Information on the STROBE Initiative is available at www.strobe-statement.org.
